# Hypoproliferative human neural progenitor cell xenografts survived extendedly in the brain of immunocompetent rats

**DOI:** 10.1186/s13287-021-02427-1

**Published:** 2021-07-02

**Authors:** Chunhua Liu, Xiaoyun Wang, Wenhao Huang, Wei Meng, Zhenghui Su, Qi Xing, Heng Shi, Di Zhang, Min Zhou, Yifan Zhao, Haitao Wang, Guangjin Pan, Xiaofen Zhong, Duanqing Pei, Yiping Guo

**Affiliations:** 1 CAS Key Laboratory of Regenerative Biology, Guangzhou Institutes of Biomedicine and Health, Chinese Academy of Sciences, Guangzhou, 510530 Guangdong Province China; 2 Guangdong Provincial Key Laboratory of Stem Cell and Regenerative Medicine, Guangzhou Institutes of Biomedicine and Health, Chinese Academy of Sciences, Guangzhou, 510530 Guangdong Province China; 3 Guangzhou Regenerative Medicine and Health Guangdong Laboratory (GRMH-GDL), Guangzhou, 510005 China; 4 Guangdong Work Injury Rehabilitation Center, Guangzhou, 510440 China; 5 Department of Biomedical Sciences, City University of Hong Kong, Kowloon Tong, Hong Kong, China; 6 CAS Key Laboratory of Brain Function and Disease, University of Science and Technology of China, Hefei, China; 7 Drug Discovery Pipeline, Guangzhou Institutes of Biomedicine and Health, Chinese Academy of Sciences, Guangzhou, 510530 Guangdong Province China

**Keywords:** Human neural progenitor cells, Xenograft, Immuno-rejection, Hypoproliferation, Immune privilege, Microglia

## Abstract

**Background:**

There is a huge controversy about whether xenograft or allograft in the “immune-privileged” brain needs immunosuppression. In animal studies, the prevailing sophisticated use of immunosuppression or immunodeficient animal is detrimental for the recipients, which results in a short lifespan of animals, confounds functional behavioral readout of the graft benefits, and discourages long-term follow-up.

**Methods:**

Neuron-restricted neural progenitor cells (NPCs) were derived from human embryonic stem cells (ESCs, including H1, its gene-modified cell lines for better visualization, and HN4), propagated for different passages, and then transplanted into the brain of immunocompetent rats without immunosuppressants. The graft survivals, their cell fates, and HLA expression levels were examined over time (up to 4 months after transplantation). We compared the survival capability of NPCs from different passages and in different transplantation sites (intra-parenchyma vs. para- and intra-cerebroventricle). The host responses to the grafts were also investigated.

**Results:**

Our results show that human ESC-derived neuron-restricted NPCs survive extendedly in adult rat brain parenchyma with no need of immunosuppression whereas a late-onset graft rejection seems inevitable. Both donor HLA antigens and host MHC-II expression level remain relatively low with little change over time and cannot predict the late-onset rejection. The intra-/para-cerebroventricular human grafts are more vulnerable to the immune attack than the intrastriatal counterparts. Prevention of graft hyperplasia by using hypoproliferative late passaged human NPCs further significantly extends the graft survival time. Our new data also shows that a subpopulation of host microglia upregulate MHC-II expression in response to the human graft, but fail to present the human antigen to the host immune system, suggestive of the immune-isolation role of the blood–brain barrier (BBB).

**Conclusions:**

The present study confirms the “immune privilege” of the brain parenchyma and, more importantly, unveils that choosing hypoproliferative NPCs for transplantation can benefit graft outcome in terms of both lower tumor-genic risk and the prolonged survival time without immunosuppression.

**Supplementary Information:**

The online version contains supplementary material available at 10.1186/s13287-021-02427-1.

## Background

It is now well accepted that the pluripotent stem cell-based cell therapy holds the promise for traditionally incurable neurological diseases. Prior to translation into human clinical trials, thorough preclinical in vivo efficacy testing of in vitro induced human neural progenitor cells has to be performed, where rodents and non-human primates are the most important and indispensable animal models. Most xenograft and allograft experiments used high doses of immunosuppressants throughout the lifespan of the animals or use immunodeficiency animals. However, overdosed immunosuppression or immunodeficiency results in the susceptibility to infections and thus short lifespan of animals, which contrasts to the prolonged time windows required by human neural cells to fully mature and integrate into host brain networks [1–3]. Even more, long-term frequent injection of animals is labor-intensive for personnel and painful and stressful for the animals, which has confounded functional behavioral readout of the graft benefit and discouraged long-term animal studies [4].

The effort to balance the cell rejection and immunosuppression to reduce the side effects has never stopped. The major route of graft rejection occurs via the recognition of foreign protein molecules (e.g., MHC molecules) by the host immune system. The rate at which heterogenous grafts are rejected depends on both MHC disparities and transplantation site [5]. Previous studies have reported that human and mouse fetal/embryonic cells, including embryonic stem cells and their derivatives, express low level of MHC-I, MHC-II, and/or co-stimulatory molecules [6–13]. And the central nervous system is a so-called immune-privileged site where the immune response to allografts is considerably restricted [14–16]. Previous studies have succeeded in transplantation of allogeneic (with animal donor cells) and xenogeneic (with animal or human donor cells) fetal/embryonic neural cells in animals without immunosuppression [17–21]. More than 20 years of clinical applications of embryonic mesencephalic allografts for the therapy of Parkinson’s disease also demonstrated a lack of detectable systemic humoral/cellular allogeneic response in human recipients under no immunosuppression condition [16, 22]. This, however, has been challenged by some experimental and clinical evidence [23–26]. A clinical study has reported that 4 in 12 Huntington’s disease patients grafted with fetal neural cells demonstrated alloimmunization and the fifth patient showed overt rejection 14 months after grafting, which is reversible with immunosuppressive treatment [26]. All these debates implied that there is still quite a lack of knowledge on the immunological graft–host interactions in the central nervous system (CNS). A more precise knowledge about the survival of human neural stem cells in animal brain without immunosuppression, and the mechanisms underlying the rejection in the CNS, may prevent unnecessarily excessive suppression of the immune system in preclinical animal studies, even in patients, warranting the present study.

## Methods

All experiments were conducted in accordance with the Guide for the Care and Use of Laboratory Animals of the National Institute of Health (Publication No. 80-23, revised 1996) and were approved by the Experimental Animal Ethics Committee at Guangzhou Institutes of Biomedicine and Health (GIBH), Chinese Academy of Sciences (IACUC NO. 2012008).

### Human embryonic stem cell culture and neural induction

Two human ESC lines, H1 (passage 60–65, Wicell, Madison, WI, USA) and HN4 (passage 20–30), were adopted in the present study. HN4 hESC line was isolated in Reproductive Medical Center of Hainan Province, Hainan Medical College (Haikou, China) and maintained in South China Stem Cell Bank, GIBH, CAS (Guangzhou, China). All these pluripotent cells were cultured on plates coated with Matrigel (BD Biosciences, San Jose, CA, USA) in mTesR1 medium (Stemcell Technologies, Vancouver, BC, Canada) and routinely passaged by EDTA (ethylene diamine tetraacetic acid, 0.5 mM) dissociation every 4–5 days.

Neural induction was performed as previously reported [27, 28], by dual inhibition of SMAD signaling with empirical modifications to get highly homogenous neural progenitor cells (NPCs) of dorsal forebrain identity. Briefly, hESCs in monolayer culture were first induced to neuroepithelia cells in N2B27 medium (DMEM/F12: neurobasal 1:1, 0.5% N2, 1% B27, 2 mM Glutamax, 1 × NEAA, 5 μg/mL insulin, 2 μg/mL heparin) containing 5 μM SB431542 and 5 μM dorsomorphin for 8 days. After passaging, the cells were cultured in N2B27 medium for another 8 days. Twenty nanograms per milliliter of bFGF was added upon the appearance of rosette-like structures at days 12–13 for 3–4 days. Then, NPCs were harvested by manually picking of rosette-like structures and propagated on Matrigel-coated plate or as suspended neurospheres in N2B27 medium without bFGF and EGF until transplantation. For early passaged NPC transplantation, NPCs within 26–56 days (passage 2–5) after the onset of neural induction were used, and late passaged NPCs were propagated more than 90 days (more than passage 8) upon neural induction.

### Animal surgery and NPC transplantation

Both male and female Wistar or Sprague-Dawley (SD) rats aged 6–9 weeks were purchased from Charles River (Beijing, China) or Southern Medical University (Guangzhou, China) and normally fed until an age of 8–12 weeks under SPF environment. Upon transplantation, rats were anesthetized via an intraperitoneal injection of 1.0–1.5% pentobarbital sodium (40–60 mg/kg body weight). Atropine sulfate (Sigma, 0.05 mg/kg, s.c.) was administrated immediately after anesthesia. The subjects were mounted on the stereotaxic device, and then, an incision was made in the scalp, a small hole was drilled over the targeting injection site, enabling us to vertically access the striatum or lateral ventricle. After thorough hemostasis, the NPCs were transplanted. Upon transplantation, the NPCs were digested into single cells with Accutase and suspended in DMEM/F12 (7.5 × 10^4^ cells/μL). 10–15 × 10^4^ cells in total were microinjected in a single site (for striatum: AP + 1.0, LM3.2, DV5.2 relative to Bregma; for deep in motor cortex: for striatum: AP + 1.2, LM2.2, DV3.2 relative to Bregma). After suture, the rats were then allowed to recover on a temperature-controlled blanket and returned to their home cages with clean beddings. Antibiotics were applied daily in the first week after transplantation. No immunosuppressant was used in the present study unless otherwise stated in control experiments.

### Brain slicing and immunofluorescence staining

Rats were anesthetized with a lethal dose of pentobarbital sodium (200 mg/kg body weight, i.p.) and perfused transcardially with 0.9% NaCl or with 0.1 M PBS followed by cold and fresh 4% paraformaldehyde (PFA) in 0.1 M phosphate buffer (pH 7.4). Brains were quickly collected, postfixed in 4% PFA overnight, and cryoprotected for 48–72 h in 30% sucrose at 4 ° . Brains were sectioned coronally on a cryostat (Leica CM3050S) at 40-μm thickness into 0.1 M PBS and processed for immunostaining.

For immunostaining, free-floating sections were washed in PBS 3 times and permeabilized with 1% Triton X-100 in PBS for 1 h, followed by incubating in blocking buffer (PBS containing 10% goat or donkey serum and 0.3% Triton X-100) for 1–2 h. Sections were incubated in the primary antibodies diluted in blocking buffer at 4 °C on a shaker overnight and then in fluorescein-conjugated secondary antibodies and DAPI for 1 h at room temperature. Three times washing with PBS (15 min for each time on shaker) followed each antibody incubation. Sections were mounted on a glass slide with an anti-fluorescence quenching mounting medium for further imaging and analysis. All images were collected on a Zeiss LSM800 confocal microscope and processed with Zen software, ImageJ software (NIH), and Adobe Photoshop CS4 (Adobe Systems, San Jose, CA).

Primary antibodies used in this study include anti-hNA (Millipore, MAB1281, 1:500), anti-hNA (Millipore, MAB1281A4,1:100), anti-IBA-1 (Wako, 019-19741, 1:1000), anti-CD3 (Abcam, ab5690,1:200), anti-DCX (Cell Signaling,1:500), anti-Map2 (Abcam, ab32454, 1:500), anti-GFAP (Dako, Z0334,1:1000), anti-Nestin (Millipore, ABD69, 1:1000), anti-NeuN (Millipore, MAB377,1:500), anti-Oct4 (Santa Cruz, sc-5279, 1:200), anti-Sox17 (R&D systems, AF1924, 1:400), anti-AFP (GeneTex, GTX84948, 1:200), anti-HLA-ABC (Abcam, ab70328,1:500), anti-HLA-DR (Abcam, ab223907,1:200), anti-Ki67 (Abcam, ab15580, 1:1000), and anti-MHC-II (Abcam, ab23990,1:100). Secondary antibodies include goat anti-rat IgG Fc secondary antibody FITC conjugate (Invitrogen, TA2505616, 1:1000), goat anti-rabbit IgG(H + L) secondary antibody Alexa Fluor 647 (Invitrogen, A32733, 1:500), donkey anti-goat IgG(H + L) secondary antibody Alexa Fluor 647 (Invitrogen, A21447, 1:500), goat anti-mouse IgG(H + L) secondary antibody Alexa Fluor 568 (Invitrogen, A11004, 1:500), and goat anti-mouse IgG(H + L) secondary antibody Alexa Fluor 488 (Invitrogen, A11001, 1:500).

### Rabies virus injection

For retrograde monosynaptic tracing, we used another genetically modified human embryonic stem cell line (H1-CAG-GTRqp) that constitutionally expresses rabies virus glycoprotein, avian TVA receptor (required for selective infection with EnvA-pseudotyped glycoprotein-deleted rabies virus (ΔGRV)) and EGFP under control of the human CAG promoter. One week before the sacrifice of the animals, rabies virus for retrograde monosynaptic tracing was injected at 0.5-mm distance above the transplantation site and rats were perfused 7 days later. The titer of the virus was approximately 1.0 × 10^7^ IU/mL tested before frozen storage at −80 °0. A pulled glass micropipette (outer diameter of tip, 30–40 μm) connected to a microinjector (Stoelting, 53311) was used for virus injection. A volume of 1.0 μL virus was injected in a single site, with a velocity of 0.1 μL/min. To reduce backflow after injection, the glass micropipette was left in place for another 10 min before slowly withdrawing.

### qPCR

Rats were anesthetized with a lethal dose of pentobarbital sodium (200 mg/kg body weight, i.p.) and perfused transcardially with cold 0.9% NaCl. Brains were quickly collected and snap-frozen in liquid nitrogen prechilled isopentane in Tissue-Tek OCT (Sakura, 4583) and transferred to a −80 °0 freezer for storage before the experiment. After carefully locating human cells via cryosectioning, a small piece of striatum tissue containing human graft core was carefully isolated under stereoscopy and homogenized in extraction buffer provided in the total RNA extraction kit (Cat#DP431, Tiangen). The total RNA was prepared according to the manufacturer’s protocol. The first strands of cDNA were prepared with FastKing RT kit (With gDNase) (Cat#KR116, Tiangen) and quantified with Talent qPCR PreMix kit (Cat#FP209, Tiangen) on the CFX-96 real-time PCR detection system (Bio-Rad). β-actin was used to normalize the measured transcript. Primer sequences are listed in the supporting information.

### Western blot

Cells or brain tissues containing the human graft were harvested and the protein was prepared with Whole Protein Extraction Kit (KGP2100, Keygen). After centrifugation at 4 °C, 12,000 rpm for 5 min, the protein content of cell lysates was determined using UV spectrophotometry (Nanodrop, IMPLEN GMBH, NanoPhotometer N90 Touch). Equal amounts (30 mg) of protein were loaded per lane and electrophoresed in a 12% acrylamide gel, which was run at 100 V for 1.5 h. Protein transfer was performed using nitrocellulose for 1.5 h at 300 mA. The primary antibodies used were anti-HLA-ABC (Abcam, ab70328, 1:1000) and anti-HLA-DR (Abcam, ab223907, 1:1000). Anti-mouse HRP and the Immobilon ECL Ultra Western HRP substrate (WBULS0500, Merckmillipore Millipore) were used to detect protein.

### ELISA

Twenty-four hours after the 2nd injection of LPS, rats were anesthetized with a lethal dose of pentobarbital sodium (200 mg/kg body weight, i.p.) and perfused transcardially with cold 0.9% NaCl. The fresh cerebral cortex and striatum tissues were isolated and homogenized in cold PBS at a fixed ratio of weight/volume and centrifuged at 13,000*g* for 10 min at 4 °C. Supernatants were used to evaluate the soluble rat IL-1β (ER008, Excell Bio) and rat TNFα (ER006, Excell Bio) according to the manufacturer’s protocols. Briefly, double-antibody sandwich ELISA was adopted in both kits.

### Data analysis

Quantitative analyses were done by an operator blind to the experiment design. For statistical analysis of the population data, three animal samples from each group were randomly selected to do immunostaining against specific markers. An unpaired t-test was used to examine the differences between two groups. Analysis of variance (ANOVA) followed by Tukey’s multiple comparison test for post hoc analyses was used to examine the differences among three or above groups. For graft survival curve comparison, a log-rank test was used. GraphPad Prism software (v6.0) was used to run the tests. Data were considered significantly different when *p* was < 0.05 (*p < 0.05; **p < 0.01).

## Results

### Human NPCs survived healthy in the brain parenchyma of adult rats without immunosuppression

To directly visualize the survival of grafts, we used 2 reporter human cell lines (H1-CAG-DsRed or H1-CAG-GFP) that were generated in our lab with constitutive expression of DsRed or GFP reporter gene, at AAVS1 locus of H1 human embryonic stem cell (hESC) through CRISPR/Cas9 gene targeting [28].

**Fig. 1:**
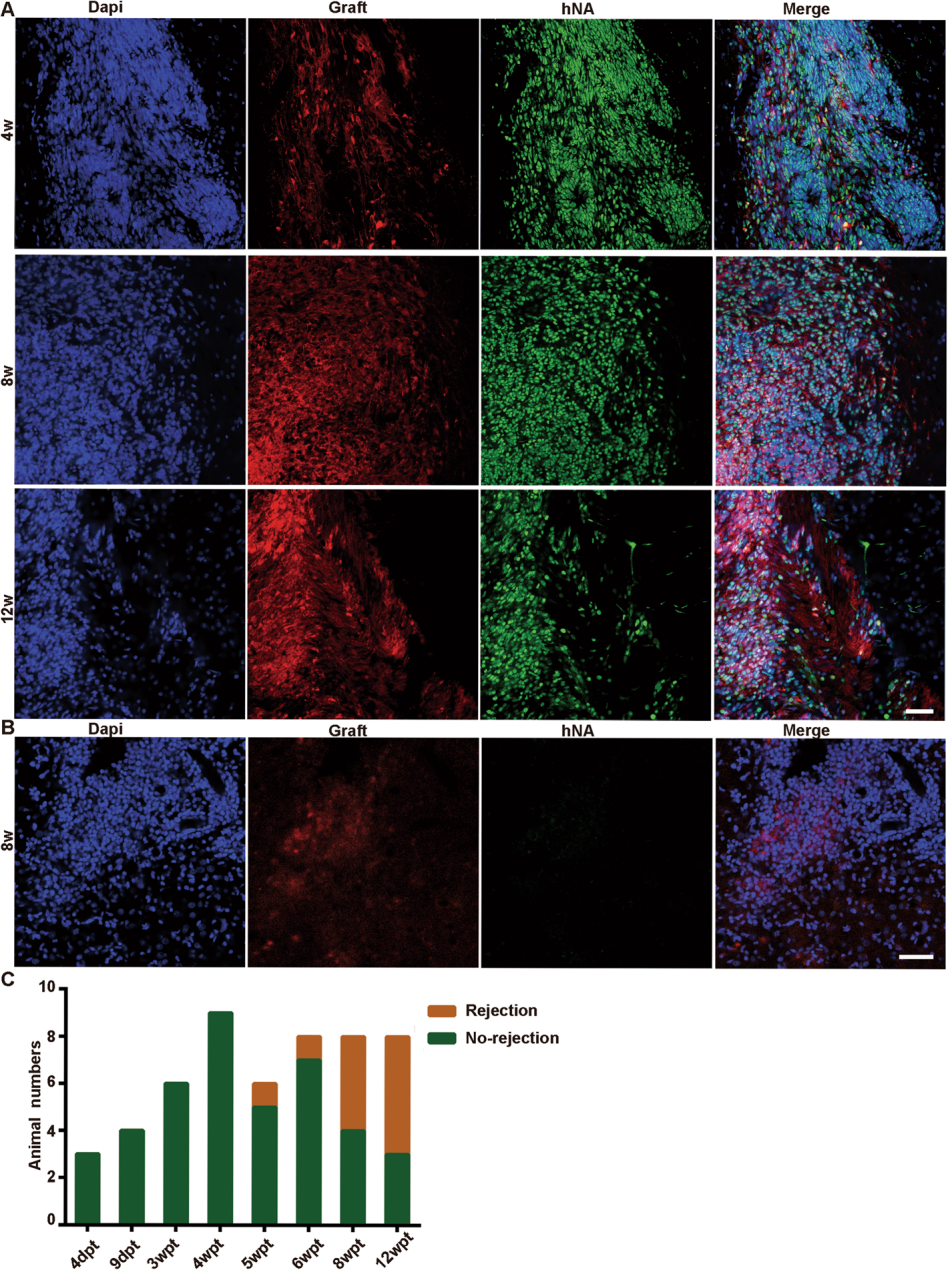
Early passaged human ESC-derived NPC grafts survive in immunocompetent adult rats up to 12 weeks post-transplantation (wpt). **A** Verification of healthy human NPCs and graft expansion by co-localization of DsRed reporter and human-specific nuclear antigen. Scale bar, 50 μm. **B** A representative picture of massive human cell death occurred at late time points in some recipients, showing lost DsRed reporter and human-specific nuclear antigen. **C** Population data showing that all human grafts survive healthily within 4 wpt, and a late-onset rejection occurs thereafter with a rejection rate more than 50% at 12 wpt for early passaged NPCs

Early passaged (P2–P5, day 26–56 after neural induction onset) human H1-CAG-DsRed-derived NPCs were transplanted into the intact striatum of immunocompetent adult rats. Within 4 weeks after transplantation, grafts survived healthy in *ALL* animals with no need of immunosuppression, and no obvious immune rejection was observed in any time point, from 4 days (dpt) to 4 weeks (wpt) post-transplantation (0/22 animals). Human cells were identified by co-localization of human-specific nuclear antigen (hNA) and DsRed reporter (Fig. 1A). The first case of massive cell death of human grafts (due to the immuno-rejection, see below) happened at 5 wpt, with a rejection rate of 15.4% (2/14) at 5–6 wpt. The rejection rate abruptly increased to more than 50% (9/16) at 8–12 wpt (Fig. 1B, C). Surprisingly, although dead cells (not unequivocally from grafted cells) might be observed in the injection track or around due to stab injury or secondary inflammation, no brains harbored both live and dead human cell mass within the graft core colony, implicating that the massive graft necrosis was a transient process and took place as an all-or-nothing event.

We repeated the experiment by using another hESC line (HN4). All rats (3/3) with intrastriatal HN4 graft had live graft colonies when sacrificed at 4 wpt, whereas overt rejections occurred in 8-wpt rats (4/4), probably due to a high proliferation rate of this batch of HN4 NPCs in a retrospective analysis (Fig. S1).

### Engrafted human NPCs had a neuron-restricted cell fate

We used early passaged NPCs to transplant in most of our experiments. After being settled down (usually within 2 wpt), these engrafted human NPCs started to expand and formed rosette-like structure at 3–5 wpt, which disappeared at longer time points. None of the host brains harbored a teratoma within the observation time window (H&E staining, Fig. S2A), as confirmed by immunostaining analysis that failed to detect any human cells positive for OCT4, AFP, and sox17, used as markers for residual pluripotent or non-neural cells (Fig. S2B). Notably, all animals lived healthily without any signs of depression or anxiety before sacrifice.

**Fig. 2:**
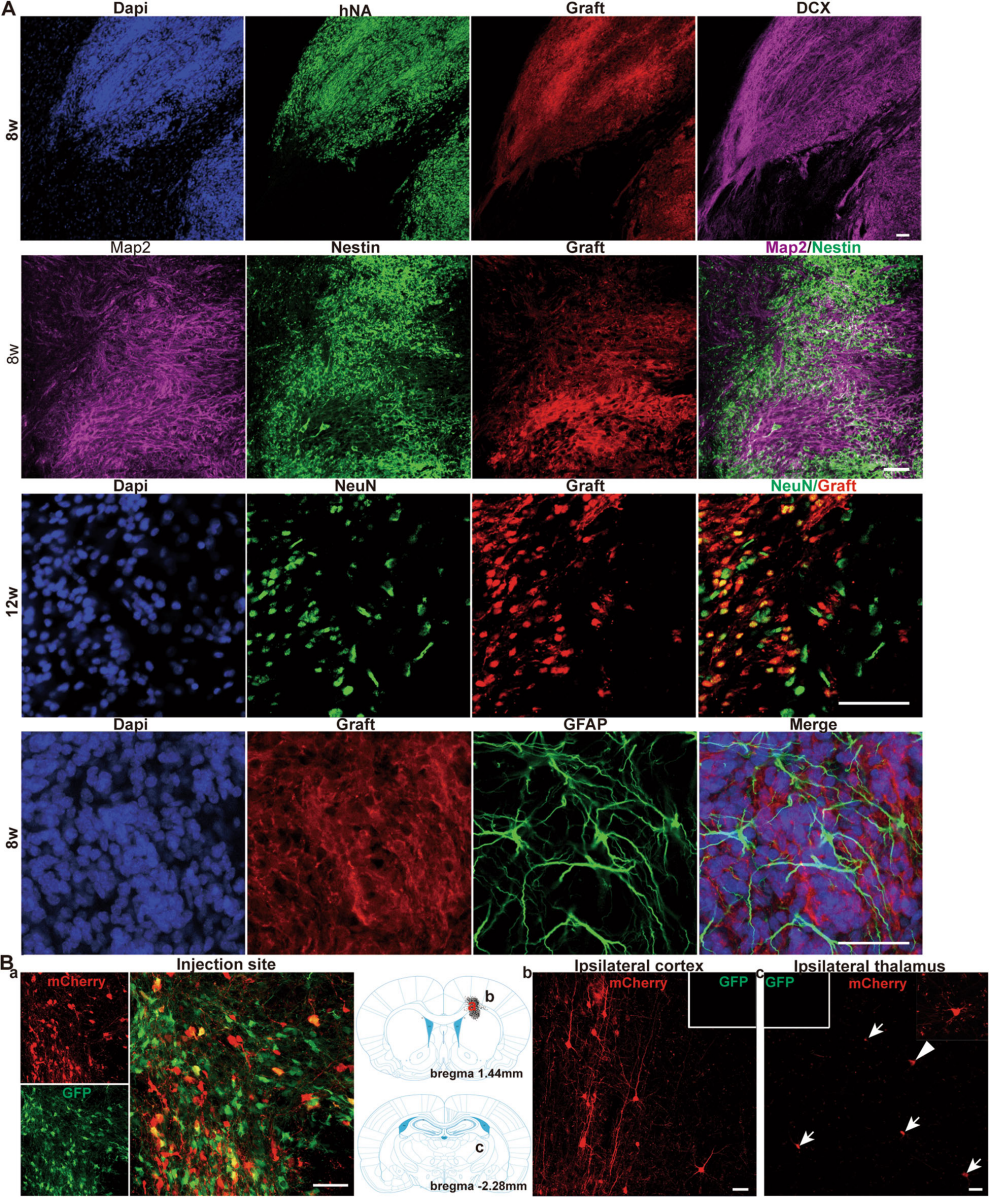
Engrafted human NPCs differentiate into neurons and further incorporate into host neural network under settings without immunosuppression. A Expressions of pro-neuronal markers (DCX, MAP2, and NeuN), but not of astrocyte marker (GFAP) within 12 weeks post-transplantation. **B** Retrograde monosynaptic tracing showing that long projection inputs from host neurons to engrafted cells originate from both the ipsilateral neocortex and thalamus. **B** (a) The co-existing of EGFP and mCherry in the starter human neurons (EGFP+mCherry+); **B** (b, c) the traced host neurons with only mCherry labeling (EGFP-mCherry+ (shown in inset), indicated by arrows and arrowhead) in the ipsilateral cortex (**B** (b)) and the ipsilateral thalamus (**B** (c)). The cell indicated with the arrowhead is magnified in the inset (upright corner). Scale bar, 50 μm

Although quite a few human cells migrated away along the white matter tracts or blood vessels (Fig. S2C), the majority of the engrafted cells stayed around the injection sites and dispersed within the vicinity, forming a graft core (Fig. 1A). In this core, a large number of cells expressed NESTIN (neural stem cell marker), and/or early neuronal cell fate-committed markers, like DCX (neuroblast marker), TUJ1 (early neuron precursor) at 3–8 wpt (Fig. 2A). Many grafted cells also expressed MAP2 (another neuron marker) during this period, while NeuN expression only manifested by 8–12 wpt (Fig. 2A), supporting the notion that a prolonged time window is needed for human NPCs to differentiate into mature neurons. We failed to detect GFAP expression in human cells at any time point up to 12 wpt (Fig. 2A), indicative of highly pure neuron-restricted human NPCs transplanted. Although we transplanted the human NPCs into the striatum, they largely expressed cortical markers as we previously reported, like TBR1 (a general cortical neuron marker but stronger in layers V and VI), Satb2 (layers II–III), and Ctip2 (layers V and VI corticospinal projection neurons, and medium spiny neurons in striatum), while human cells with clear GAD67 expression were also found in clusters (Fig. S3).

We further investigated whether the human neurons could incorporate into host neural circuits without immunosuppression by using retrograde monosynaptic tracing we recently reported [28]. We used another genetically modified hESC line (H1-CAG-GTRqp) that constitutionally expresses rabies virus glycoprotein, avian TVA receptor (required for selective infection with EnvA-pseudotyped glycoprotein-deleted rabies virus (ΔGRV)) and EGFP under control of the CAG promoter. At 11 wpt, rabies virus (ΔGRV) was injected at 0.5-mm distance above the transplantation site (deep in motor cortex) and rats were perfused 7 days later. The infected human cells co-labeled by EGFP and mCherry (EGFP+/mCherry+, Fig. 2B (a)) worked as the starter neurons, which received the afferent projections from the host neurons that are only showing mCherry positive (as the traced neurons, EGFP−/mCherry+) in both the ipsilateral cortex (Fig. 2B (b)) and the ipsilateral thalamus (Fig. 2B (c)).

All these data showed that the human NPCs were highly neuronal-restricted and could migrate, differentiate into neurons, and further integrate into the host neural network under no immunosuppression settings.

### Late-onset cell death was due to immune rejection

**Fig. 3:**
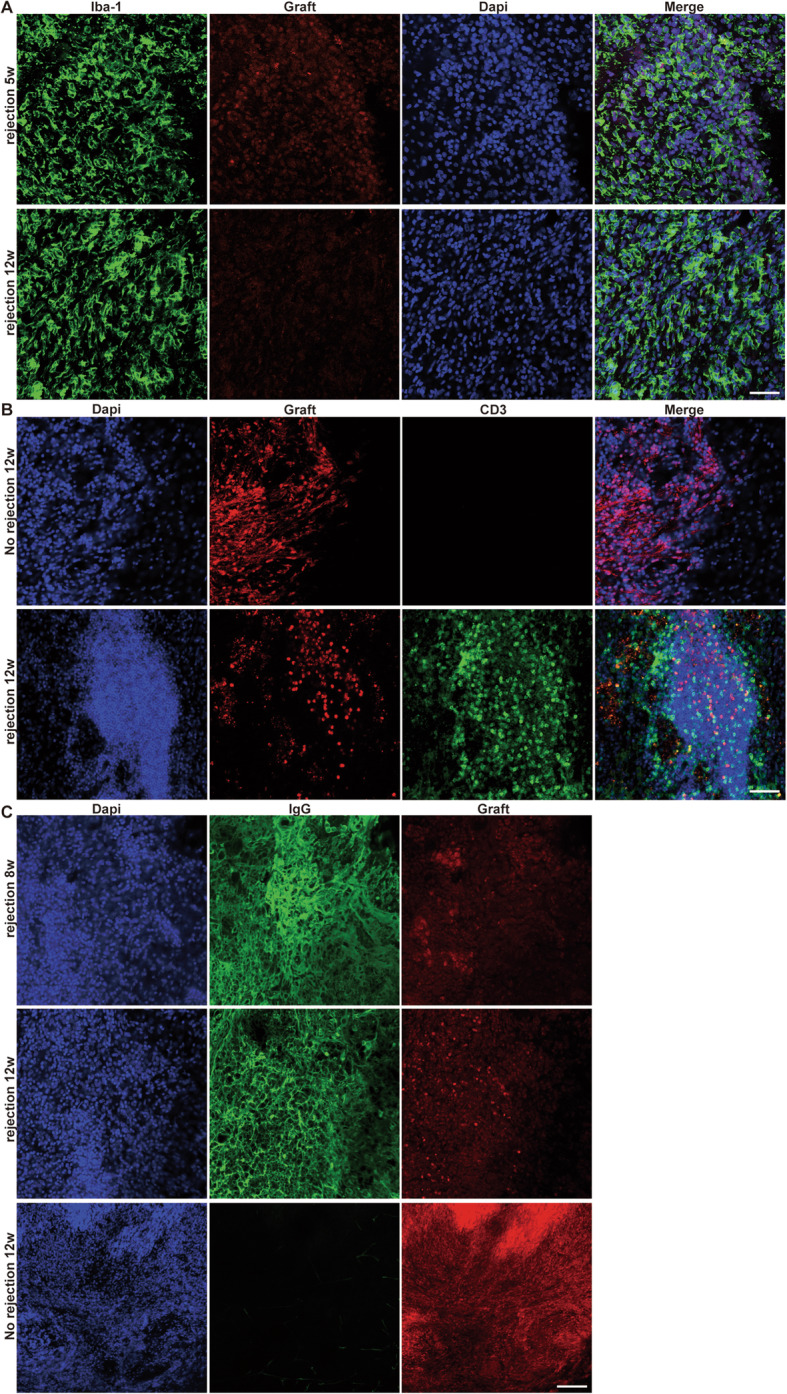
The massive death of human NPC grafts is due to immune rejection in the vast majority of cases. **A** The reoccupation of the rejected graft area by the host cells with strong IBA1 expression. Scale bar, 50 μm. **B** The infiltration of the rejected grafts with CD3+ lymphocytes which are completely absent in non-rejected human grafts. Scale bar, 50 μm. **C** Heavy host IgG deposition within the rejected grafts but not in the non-rejecting brain. Scale bar, 50 μm

As mentioned above, at late time points (i.e., 5, 6, 8, and 12 wpt), only massive dead human cell remnants were observed in some recipients, as evidenced by TUNEL assay (Fig. S4) and dusty DsRed reporter signals (Fig. 1B) without cell morphology, where cavitation and cracks were sometimes conspicuous (Fig. S5A and Fig. S5B). The hNA-positive immunoreactivity was also found desperately messy and weak, indicative of rapid loss of cellular components of engrafted cells (Fig. 1B, also see in TUNEL assay, Fig. S4B-b). Furthermore, the graft colony was reoccupied by host cells, many of which with irregular cell body showed robust IBA1 expression, a marker of microglia or monocytes, implicating undergoing destructive phagocytosis of the dead graft cells by the host (Fig. 3A).

To figure out whether this seemingly spontaneous late-onset cell death was due to adaptive immuno-rejection, lack of oxygen and glucose supply, or other reasons, e.g., spontaneous apoptosis, we further performed H&E staining and CD3 immunostaining, a T-lymphocyte marker. In H&E staining, the rejected grafts were characterized with dense and clustered leukocyte infiltration (Fig. S5B), and many of them were CD3 positive with lymphocyte morphology (Fig. 3B). Perivascular lymphocytic cuffing was present within the graft and at the graft–host interface. We also detected heavy host IgG deposition within the rejected graft, suggestive of the precipitation of humoral immunity (Fig. 3C). Moreover, the intense infiltration of host inflammatory cells (microglia and T-lymphocytes) and IgG deposition was circumscribed to necrotic graft remnants and largely spared the neighboring host structures. In contrast, non-rejected human grafts were completely devoid of any leukocyte infiltration and IgG deposition (Fig. 3B, C).

In another experiment, we transplanted human NPCs into two different parenchymal sites of the same rats with an interval distance of 7 mm (*n* = 12) and examined at 5 wpt. Human grafts were found dead at both sites in 4 recipients, while others only harbored live human graft colony (Fig. S6). This “Both-or-None” phenomenon ruled out the possibility that the elimination of human cells was due to the lack of oxygen and glucose supply or spontaneous apoptosis. It is highly likely that hosts sensitized by either graft would launch an immune attack on both.

Taken together, our results suggested that immuno-rejection might largely account for the late-onset death of human grafts. This conclusion is also supported by our another finding that human NPC grafts survived in all recipients sacrificed at 3 and 6 mpt when daily cyclosporine immunosuppression was used [28].

### HLA-ABC and HLA-DR antigens were NOT the trigger for the late-onset rejection of human NPCs

Studies have demonstrated that MHC expression are of prime importance in allograft rejection and may be the precedent step to xenograft rejection [29, 30]. Besides HLA-ABC, HLA-DR has also long been strongly associated to human transplant rejection [31]. Human breast cancer cells (MDA-MB-231) presented relatively high-level HLA-ABC (Fig. S7A) and HLA-DR (Fig. S7E) and were quickly rejected after transplanted into the striatum (Fig. S7A). Consistent with previous studies, human ESC-derived NPCs displayed relatively low HLA-ABC and HLA-DR expression levels (Fig. S7B and S7E-F), which might protect them from immune attack at the time of graft when the blood–brain barrier (BBB) was transiently disturbed. More importantly, the HLA-ABC and HLA-DR expression remained barely detectable in non-rejected NPC grafts up to 12 wpt (Fig. S7C and S7E-F), implying that HLA-ABC and HLA-DR antigens should not be responsible for the initiation of the late-onset human cell rejection.

### Intra-cerebroventricular human NPC grafts were more vulnerable to immune attack than the intrastriatal counterpart

Notably, in some rejecting cases, we noticed that the grafts protruded into the paraventricular area, where human cell antigen might more easily get access to the immune system and trigger the immune reaction. Recent findings have revealed that circumventricular organs, such as the choroid plexus, work as gateways in the trafficking of peripheral leukocytes to the CNS [32, 33]. The meningeal lymphatic vessel was found to drain cerebrospinal fluid directly into cervical lymph nodes [34]. So, we hypothesized that the rupture of the paraventricular structure should be detrimental for survival of human grafts. If this is the case, the intra-cerebroventricular (ICV) human grafts should not be able to survive long in immunocompetent rats. Then, we transplanted human NPCs into the lateral ventricle to examine whether the cerebroventricles are immune-privileged as is the parenchyma. Four weeks later, no live graft was visualized in all these animals (*n* = 6). The ventricles were not enlarged, and only trace of dead cells sparsely scattered in the ventricles, indicative of quick depletion of human cells. Given that the ventricle of rat and mouse brain has been shown to be a preferred site for neural grafts in immunodeficient xenogeneic hosts [35], the death of intra-cerebroventricular graft was unlikely due to non-immunologically environmental factors. More importantly, the ICV graft also induced death of human NPCs deposited on the upper and lower banks of the ipsilateral lateral ventricle or in the fimbria of the hippocampus with T-lymphocyte infiltration. To further ascertain this, we injected human NPCs into a proximal site to the lateral ventricle (within 0.5 mm to the lateral ependymal wall) and found no human grafts survived at 6 wpt (*n* = 5). These results suggested that the rupture of the paraventricular structure was detrimental for the graft survival in immunocompetent rats.

### Hypoproliferative human NPC grafts further extended their survival

**Fig. 4:**
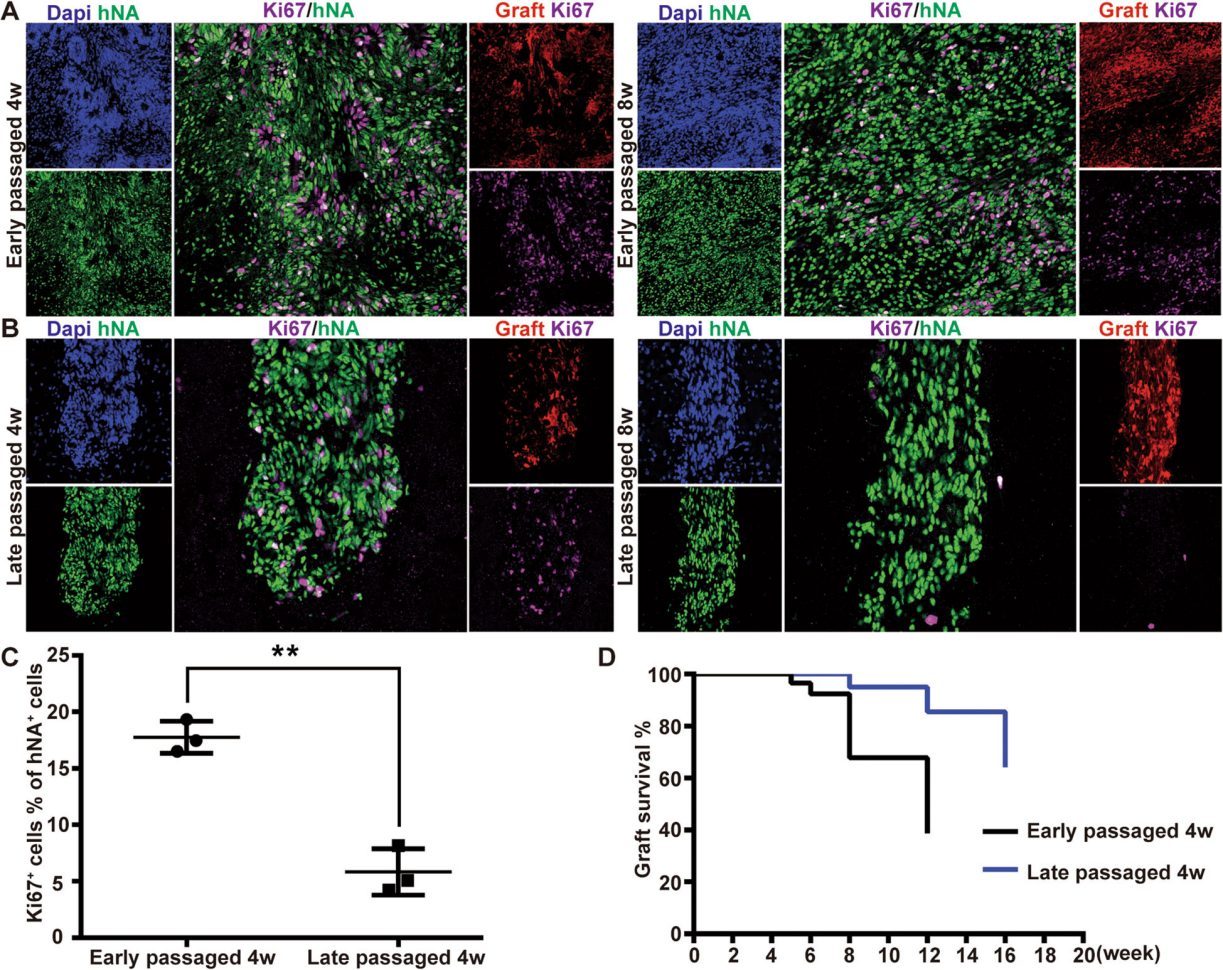
Late passaged human NPCs with low proliferation survive much longer than early passaged NPCs. **A**, **B** Ki67 immunostaining showing relative high proliferation of early passaged NPCs (**A**) and very limited proliferation of late passaged NPCs (B) at 4–8 wpt. Scale bar, 50 μm. **C** Statistical analysis of proliferation levels of early and late NPCs at 4 wpt (unpaired T-test, *P* < 0.01**). *N* = 3 biologically independent samples. **D** Comparative analysis of graft survival time of early and late passaged NPCs (log-rank test, *P* < 0.01**)

Low immunogenicity of human NPC grafts (with low HLA-ABC and HLA-DR expression level) could protect them from the host immune attack. Another arm of the CNS immune privilege is the BBB, which greatly protects the CNS from the immune attack [5]. The late-onset human graft rejection could be also due to the compromise of this barrier, e.g., the rupture of the paraventricular structure. Early passaged human NPCs started significant expansion after settled down in the rat brain and preferred to form an ever-increasing and incongruous hyperplastic core within a short time (normally within 2 months, Fig. 1 and other figures). They might destruct the local vascular system or break into the paraventricular organ via imposing a continuous compression (mechanically) or other impacts (e.g., via secreting cytokines or neuropeptides [36]) on the surrounding tissues, resulting in intermittent perturbations of local BBB or ventricle-meningeal lymphatic system. The heavy T-lymphocyte infiltration and IgG deposition within the rejected grafts did demonstrate such a vascular or paraventricular damage, which was confirmed by the immunostaining against vascular markers showing the collapse of the local vessels (Fig. S8). Thus, prevention of graft hyperplasia might be a way to avoid the graft rejection. We turned to transplant late passaged NPCs (beyond 90 days after neural induction onset), which exhibited a much lower proliferation capability (Fig. 4A–C). Even better than expected, the late human NPCs survived in the overwhelming majority of animals up to our longest survival time (2/2 at 4–5w, 9/10 at 8 wpt, 5/6 at 12 wpt, and 3/4 at more than 4 months post-transplantation) without massive cell death. Graft survival analysis showed that late passaged NPCs with low proliferation survived significantly longer than the early passaged NPCs (Fig. 4D, log-rank test, *P* < 0.01**). The late hypoproliferative NPC grafts formed a much smaller graft core than the early passaged graft at 4 wpt and thereafter (Fig. 4A vs. B). Moreover, the long-term survived human grafts migrated along the white matters into a wide territory of the ipsilateral cerebral cortex without conspicuous graft cores (Fig. S9).

However, further studies are needed to demonstrate that such a disruption of BBB or paraventricular structure occurs prior to the cell rejection, and to determine what antigens on the human cells would trigger this immune reaction in that the expression level of HLA-ABC and HLA-DR antigen remained barely detectable.

### The host microglia responded moderately to human NPC grafts

**Fig. 5:**
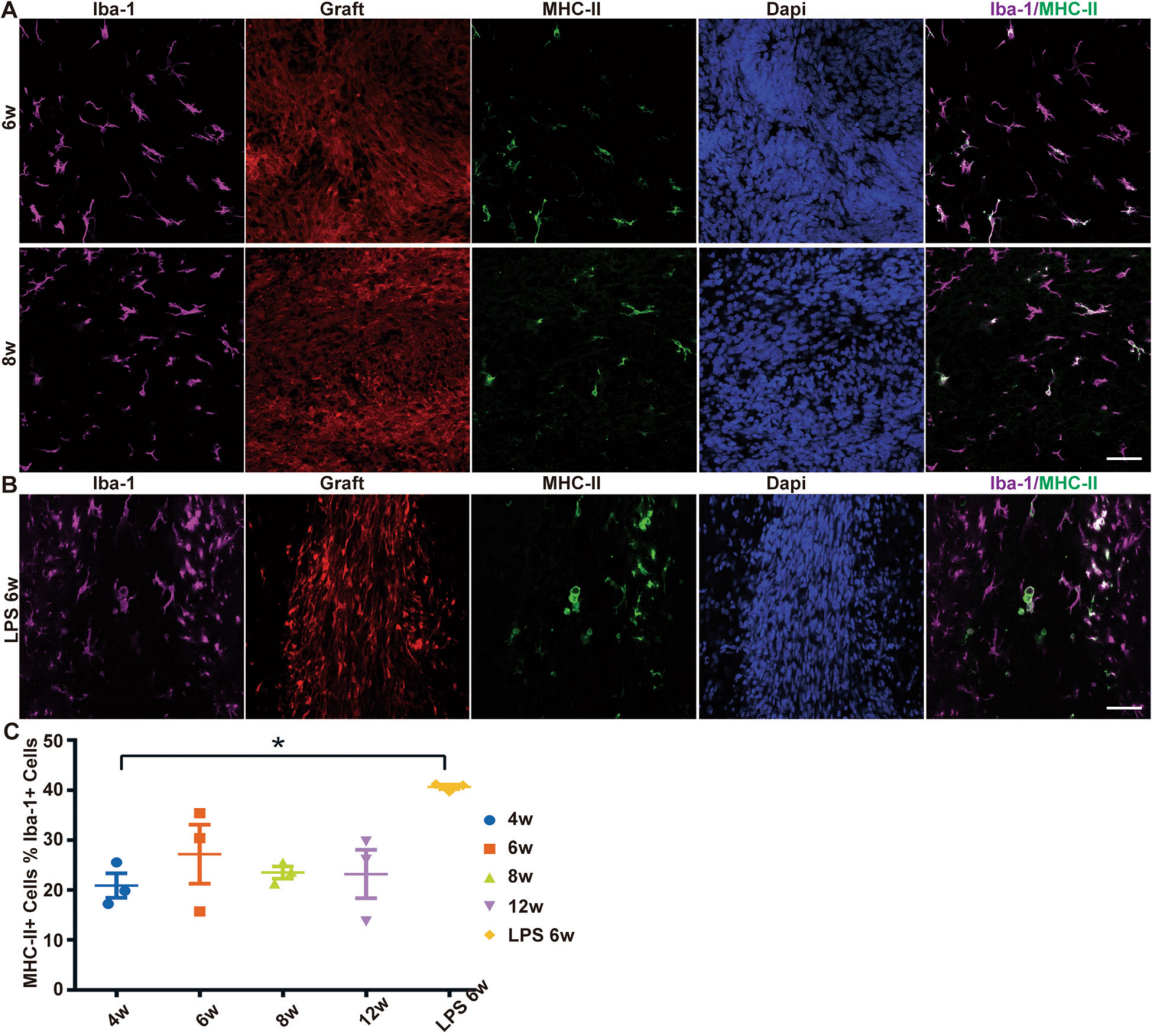
Representative pictures show a subpopulation of microglia that upregulate MHC-II expression in response to the surviving human graft. **A** MHC-II upregulation specifically observed in microglia. **B** The enhanced MHC-II expression by LPS challenge. **C** Population data showing that the host MHC-II expression remains relatively low level with no changes over time (ANOVA, *P* > 0.05). *N* = 3 biologically independent samples. Scale bar, 50 μm

Previous studies have suggested important roles of microglia in both the antigen presenting and immune attack phases of intracerebral allograft rejection [37, 38]. In the absence of CNS inflammation, microglia showed resting ramified morphology characterized by non-overlapping long branching processes and a small cellular body (Fig. S10A). One of the characteristics of microglia in vivo is their ability to surveil the parenchyma from their static tiling position [39] and to react quickly to even small pathological changes. In response to the human grafts, the host microglia proliferated, migrated into, and recolonized the graft area, where host cells including microglia had been squeezed away by the enlarged human graft (Fig. 5). Although individual phagocytic microglia were occasionally observed within the graft, most of the invading microglia showed a migrating rod-like or “cup” shape, or mildly activated with couples of thick processes (Fig. 5). This quite differed from their resting ramified morphology in the intact host brain region (Fig. S10A) and also sharply contrasted to the massive end-stage phagocytic microglia in the rejecting region, which showed intensive activation typically with hypertrophic cell body, ring or irregular shape, and severely retracted branches (Fig. 3B, Fig. S10D).

We further examined the host MHC-II expression in the non-rejecting animals to seek any early cues underlying the late-onset immuno-rejection. We failed to find any MHC-II expression in astrocytes and endothelial cells in the brain parenchyma, but a subpopulation of microglia within a healthy graft area upregulated MHC-II expression (Fig. 5A). Except for a few phagocytic microglia, nearly all MHC-II-positive microglia were mildly activated, while the resting microglia did not show detectable MHC-II expression. Notably, the host MHC-II level remained relatively low and showed no significant increase over the long-term surviving period (Fig. 5C), implying that the immuno-recognition if existed was tolerable and the MHC-II-positive microglia failed to present antigens to the host immune system and trigger downstream responses, suggestive of an immune-isolation role played by the intact BBB/ependymal wall and thus the “immune privilege” of CNS. Similarly, as mentioned below, although lipopolysaccharide (LPS) challenge further upregulated MHC-II expression around the graft moderately (Fig. 5B), it did not exacerbate the rejection of hNPC grafts.

These results proposed that the mild elevation of MHC-II expression in the microglia in response to the live human grafts may be a sign of exogenous graft-induced inflammation or the initial immuno-recognition without downstream immune reaction. The chronological quantification of MHC-II expression showed no gradual signs indicating initiation of rejection, again supporting the notion that the immuno-rejection was an unexpected and sudden event.

### LPS-induced peripheral inflammations did not increase the rejection of human NPC grafts

To test whether or not peripheral inflammation would induce the rejection of the stable human NPC grafts, we used a well-established LPS challenge paradigm [40]. Rats were injected intraperitoneally with 2.0 mg/kg LPS for 2 consecutive days at 4.5 wpt when human grafts had settled down. Systemic LPS challenge quickly induced typical inflammation symptoms (e.g., fever, loss of body weight, anorexia, and hypokinesia) and significantly upregulated the secretion of inflammation cytokine IL-1β in the brain 24 h later (Fig. S11). At 6 wpt (10 days after the second LPS injection), microglia activation could be discerned from the IBA1 immunostaining by showing mildly activated profiling with retracted processes in a brain-wide manner, as well as a brain-wide but sparse MHC-II upregulation, indicative of an undergoing inflammation (Fig. S10B-C, Fig. S12). LPS also stimulated more phagocytic microglia within the graft colony, characterized by a circle or ring shape and upregulated MHC-II expression (Fig. 5B). Nevertheless, the LPS-induced peripheral inflammation and microglia activation did not increase the rejection rate of human grafts (1/5 in LPS-challenged vs***.*** 1/6 in non-LPS). Consistently, we also did not see upregulated lymphocyte infiltration in the LPS-challenged non-rejecting brains.

## Discussion

In general, our present results confirmed the “immune privilege” of the brain parenchyma behind the BBB and ependymal wall that could substantially protect human ESC-derived NPC xenograft in the brain from immuno-rejection, much more reliably than expected. Specifically, hESC-derived NPCs survived extendedly in adult rat brain parenchyma without immunosuppression, but not in the cerebroventricles and their surrounding structures. And the hypoproliferative late passaged human NPC grafts further extended their survival (for more than 4 months), most likely by lowering the damage risks to the integrity of the BBB and ependymal wall.

Neonatal rodents have long been proven to be ideal recipients for allogenic neural grafts in the CNS with no need of immunosuppression, where the majority of allogenic grafts (globally 80% [41, 42]) could survive up to several months to even 1 year. This has been ascribed to the consensus that the injection-damaged BBB would restore before the immunological competence establishment after birth. Long-term allogenic grafts were also reported in adults with transient immunosuppression that exceeds the time necessary for the blood–brain barrier to reform [43–45], or even without immunosuppression [18, 46]. All these results suggested a critical role of intact BBB in the prevention of graft rejection. However, now most studies have been using lifelong immunosuppression or immunodeficiency animals, showing that CNS immune privilege is not absolute and immunosuppression is necessary.

### Prevention of graft hyperplasia would benefit the long-term survival of the allogenic NPC grafts

Whether the transplants rejected or not depends on both the graft immunogenicity and the host immunological status. Many variables contribute to these two factors. It is really difficult to find out where the seemly contradictory conclusions among different studies come from by comparing all these variables side by side, especially some of the details unavailable in the publications. Nevertheless, it will be much easier to tell, in retrospect, how the allogenic grafts behave under no or transient immunosuppression conditions, to avoid rejection from the published data. Notably, the long-term grafts in the neonatal brain without immunosuppression always integrated substantially with the host neural system, where large hyperplastic core rarely reported, thus remaining un-recognized or un-attacked by the host immunity system outside BBB, whereas the graft hyperplasia was quite common in the injection site of the adult recipients [1, 28]. In light of the present data, we proposed that the perinatal developmental niche, in which neural graft could better migrate, differentiate, and incorporate, might account for the long-term allogenic graft survival. In our study, the early passaged human NPCs proliferated significantly and greatly outnumbered the injected cells. These cells sometimes showed limited dispersion and deposited as an ever-increasing and dense core within a short time. The immune rejection was largely relieved when the late passaged NPC was used, where only limited cell depositions or small cell clusters were observed at 4 wpt and thereafter. Furthermore, the long-term surviving human grafts showed wide migration into the host brain territory without conspicuous graft core formed. Lund etc. also reported that smaller transplants showed significantly less lymphocytic infiltration than large ones [41]. Björklund previously hypothesized that extensive cell migration may be the key to the survival of dopaminergic xenografts in adult PD rats [18]. Daniloff and colleagues also demonstrated that a permanent incorporation of the transplanted cells into the host would be important for the long-term survival of xenograft in adult rats of the denervated hippocampus [19]. In both studies, although the bulk of the mouse graft in the rat recipient disappeared or was resorbed, the surviving neurons had migrated away from the injection site after 6 months and 17 weeks, respectively, with dense innervations to deafferented host targets. All these results implied that the potential to form hyperplastic foci was detrimental to the graft survival, and allografts in the corpus callosum or denervated brain regions could benefit from their migration or integration to avoid rejection [43, 45].

### The role of microglia in graft rejection

The presence of BBB and the lack (or low level) of cells within the brain expressing major histocompatibility complex (MHC) antigens had been thought to play key roles in maintaining CNS immune privilege. However, it has become increasingly apparent that neither factor is absolute. Increased MHC antigen expression has been demonstrated in brain injury and diseases, as well as in response to neural allograft [23, 35, 38, 47, 48]. Studies have proposed microglia as the antigen presenting cell candidate in CNS by showing a correlation between high MHC-II expression level in microglia and graft rejection, other than showing a convincing causal relationship between them [37, 47, 48]. Lawrence etc. reported that microglia were involved in both the immune presenting and immune attack phases of the intracerebral allograft rejection [37], by showing that MHC-II *gradually* upregulated in microglia in response to the allograft of embryonic hippocampal primordia. We also unveiled that the MHC-II upregulation was restricted to microglia. However, the MHC-II level herein only elevated in a subpopulation of microglia and retained low with little change over time. This discrepancy might come from different cell components of the grafts we used. Hippocampal primordia graft consisted of a variety of cell types, including vascular endothelial cells and glial cells, which acquired MHC-I and MHC-II immunoreactivity shortly in the damaged xenogeneic environment and boosted the MHC-II expression of host counterparts [37]. In contrast, our highly neurogenic NPC graft sustained a low level of HLA-ABC and HLA-DR antigens, and the host MHC-II expression only upregulated in a subpopulation of microglia, which is more likely indicative of an undergoing graft-induced low-level and tolerable inflammation. Lawrence and colleagues could also over-claim the antigen presenting role of microglia, as mentioned by themselves that MHC-II expression in the microglia was less intense than that in the endothelial cells or perivascular cuff, and the initial reactive difference between the allogenic and syngeneic grafts was related to the blood vessels. The donor endothelial cells expressing both MHC-I and MHC-II might present their own alloantigen directly to the circulating host cells after incorporated into the local vasculature. The strong phagocytosis executed by microglia and/or monocytes in the rejecting brains could also be the result of rejection as a housekeeper to clean up damaged cells and cellular debris rather than the cause as a cell killer. Therefore, it would be of great interest for us to further investigate whether the deletion of microglia could largely extend the survival of allogenic grafts.

### Implications for future preclinical studies and clinical translations

Xenograft models are very important for basic research and preclinical studies that are using human cells for transplantation. Our present results suggested that Wistar and SD rats, when used as the brain parenchymal xenograft recipients, could be taken as immune-deficient animals, with no need for immunosuppression. Although we only tested human ESC-derived NPCs, this result might be further generalized to donor cells of different species or from different sources [49]. The prerequisite includes a low MHC expression level of the donor cells and a low inflammation level when transplanted. Otherwise, such as in brain injury, either transient immunosuppression exceeding the time needed for BBB restoration should be considered [43, 45, 50] or administration of anti-inflammation reagent, such as NSAIDs, would be sufficient to avoid rejection [51].

Furthermore, peripheral inflammation would not induce the rejection of the stable human NPC grafts, even when the CNS microglia are activated with upregulated MHC-II expression. Our present data provides a good explanation for the clinical findings in more than 20 years of cellular transplant experience that autopsy of Parkinson’s disease patients with allogenic fetal neural grafts showed little immunological reactions without immune suppression.

## Conclusions

Our data proved that the “immune privilege” of the central nervous system could substantially protect human ESC-derived NPC xenograft from immuno-rejection in the rat brains. This CNS graft tolerance is not due to the anti-human-specific T cell clonal deletion [52], but largely to the immune-isolation behind the BBB and ependymal wall. No remarkable and gradual buildup of donor and host MHC levels prior to the rejection is observed, nor is the inflammation level. Choosing hypoproliferative NPCs for transplantation can benefit graft outcome in terms of a lower tumor-genic risk and the prolonged survival time without immunosuppression.

However, when coming to clinical translation, our current study shows limitations, including the relatively short observation time window post-transplantation and only rats used as subjects. Accordingly, large-animal models and a prolonged postgrafting survival beyond half a year or even more than 1 year are needed to confirm a permanent human ESC-NPC engraftment in xenogeneic settings.

## Supplementary Information

**Additional file 1.** Supplementary Figs. S1–S12.

## Data Availability

All the data supporting the conclusions of this article is included within the article and the supplemental materials.

## Abbreviations

ESC: Embryonic stem cell
NPC: Neural progenitor cell
LPS: Lipopolysaccharide
BBB: Blood–brain barrier
MHC: Major histocompatibility complex
CNS: Central nervous system
HLA: Human leukocyte antigen
H&E: Hematoxylin and eosin
hNA: Human-specific nuclear antigen
