# Supplementary material for: Hypoproliferative human neural progenitor cell xenografts survived extendedly in the brain of immunocompetent rats
Source: Stem Cell Res Ther. 2021 Jul 2;12:376. doi: 10.1186/s13287-021-02427-1 (PMC8254296; doi:10.1186/s13287-021-02427-1)
Supplement: Supplementary file 1 — Additional file 1. Supplementary Figs. S1–S12. [file 13287_2021_2427_MOESM1_ESM.pdf]

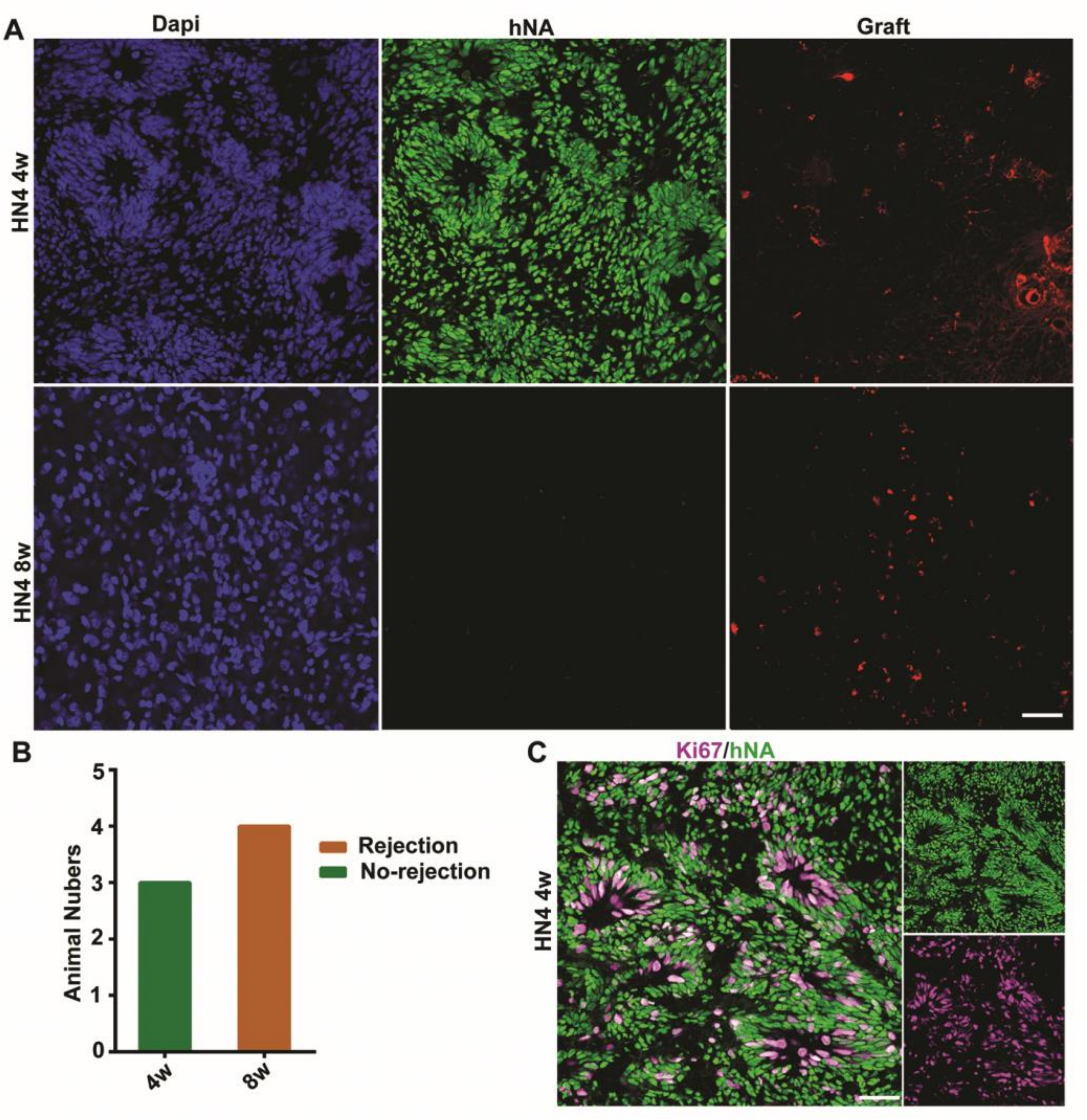

Figure S1. All NPC grafts from another Human ESC line (HN4) survive in immunocompetent adult rat brain at 4 wpt but not at 8 wpt(A-B), indicating a late-onset sudden rejection occurred. Cells were labeled with DiI (red fluorescence) before transplantation. C, relative high proliferation rate of this batch of HN4 NPCs, indicated by Ki67 staining. Scale bar, 50 μm.

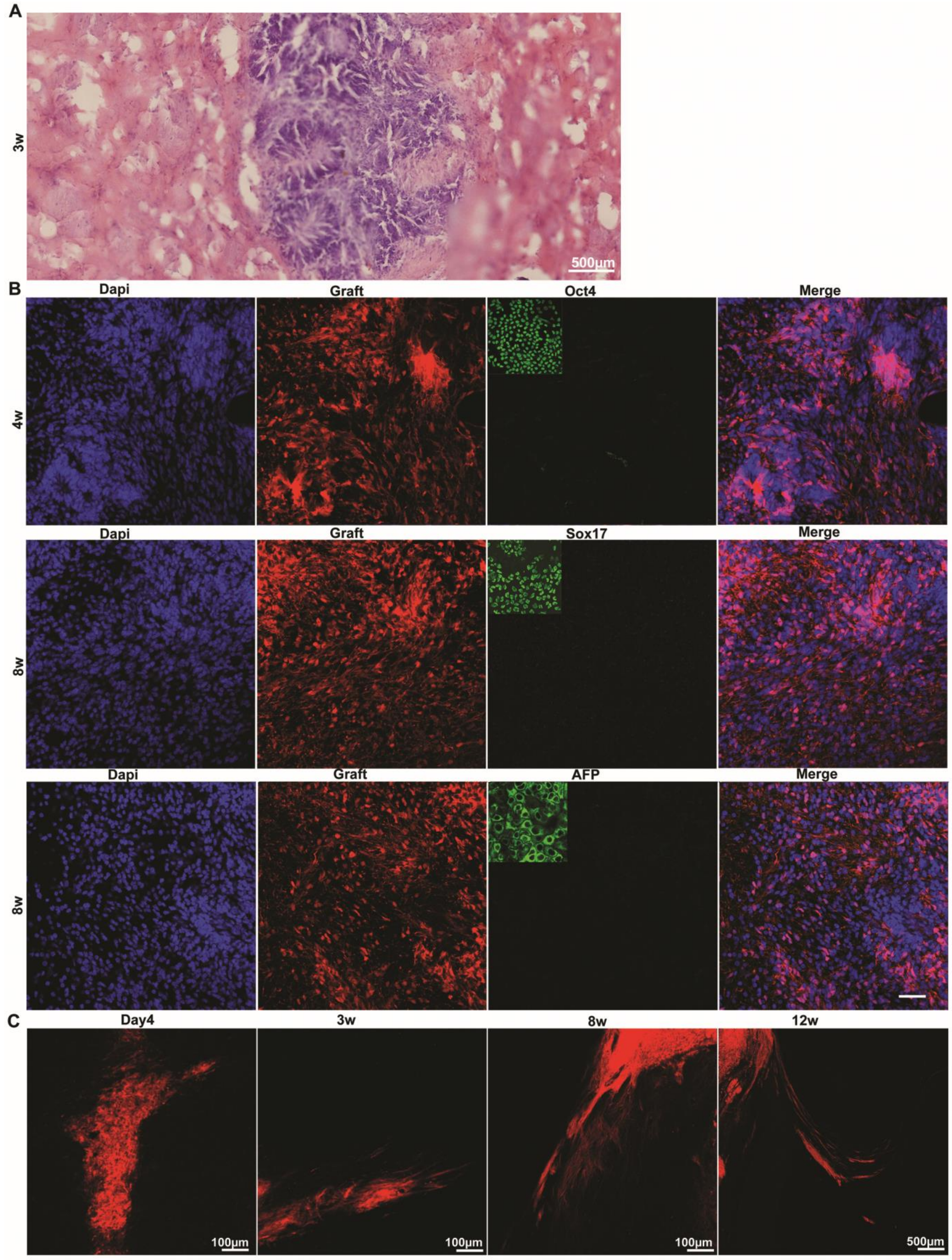

Figure S2. Cell fates of human ESC-derived NPCs in vivo, related to Figure 2. A, H&E staining showing the rosette-like structure formed by human grafts. B, No pluripotency markers (e.g. Oct4) and other non-neural markers (e.g. Sox17 and AFP) observed in human cells. Insets showing the positive control. Scale bar, 50 μm. C, well survived H1-DsRed NPC grafts showing gradual migration along white matter over time.

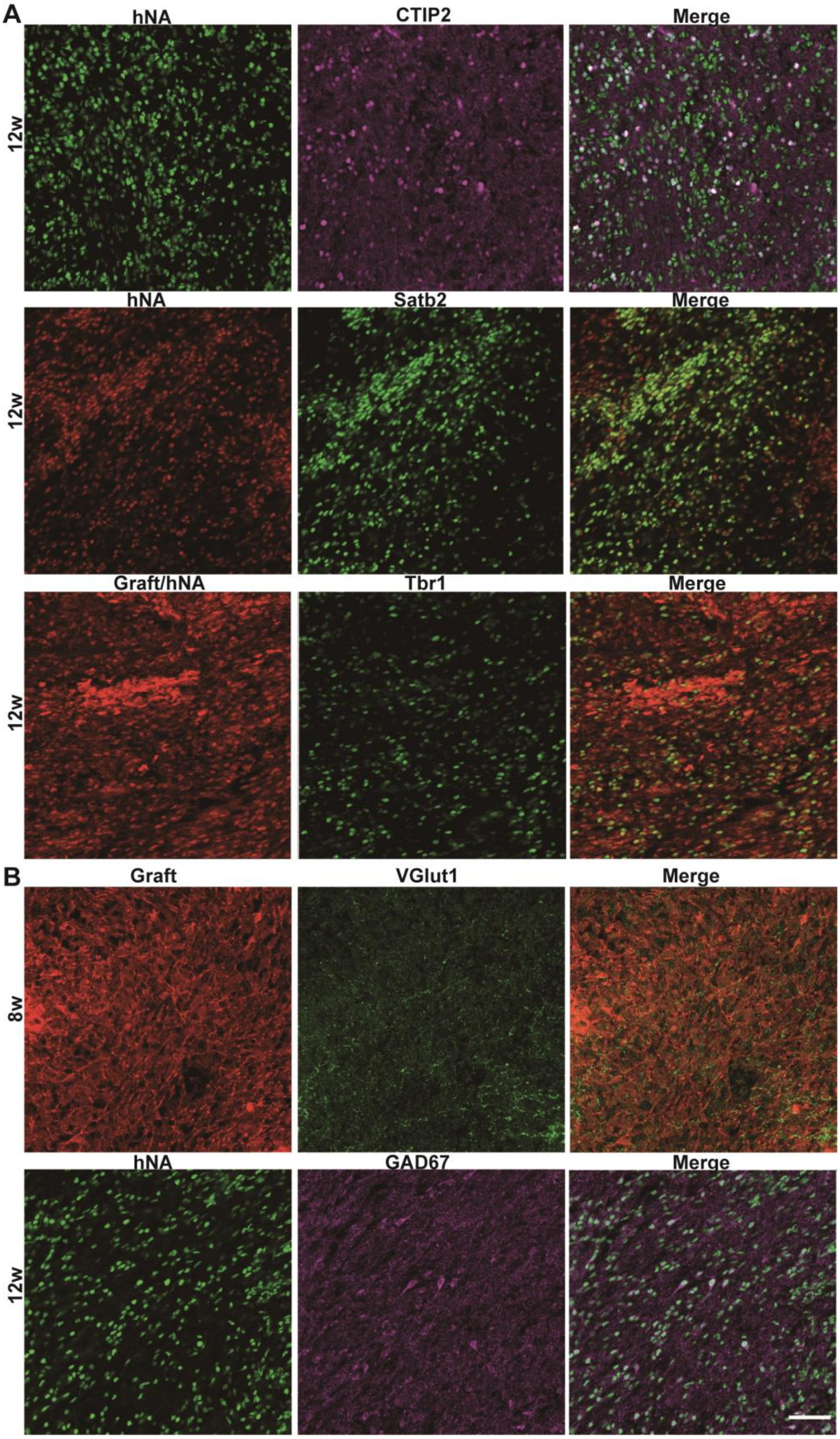

Figure S3. the intrastriatal human NPCs largely retain a cortical neuron. A, expression of cortical layer markers, like Tbr1, Satb2 and Ctip2. B, vGlut1 even expression intermingled with clustered GAD67 expression in human cells. Scale bar, 50 µm.

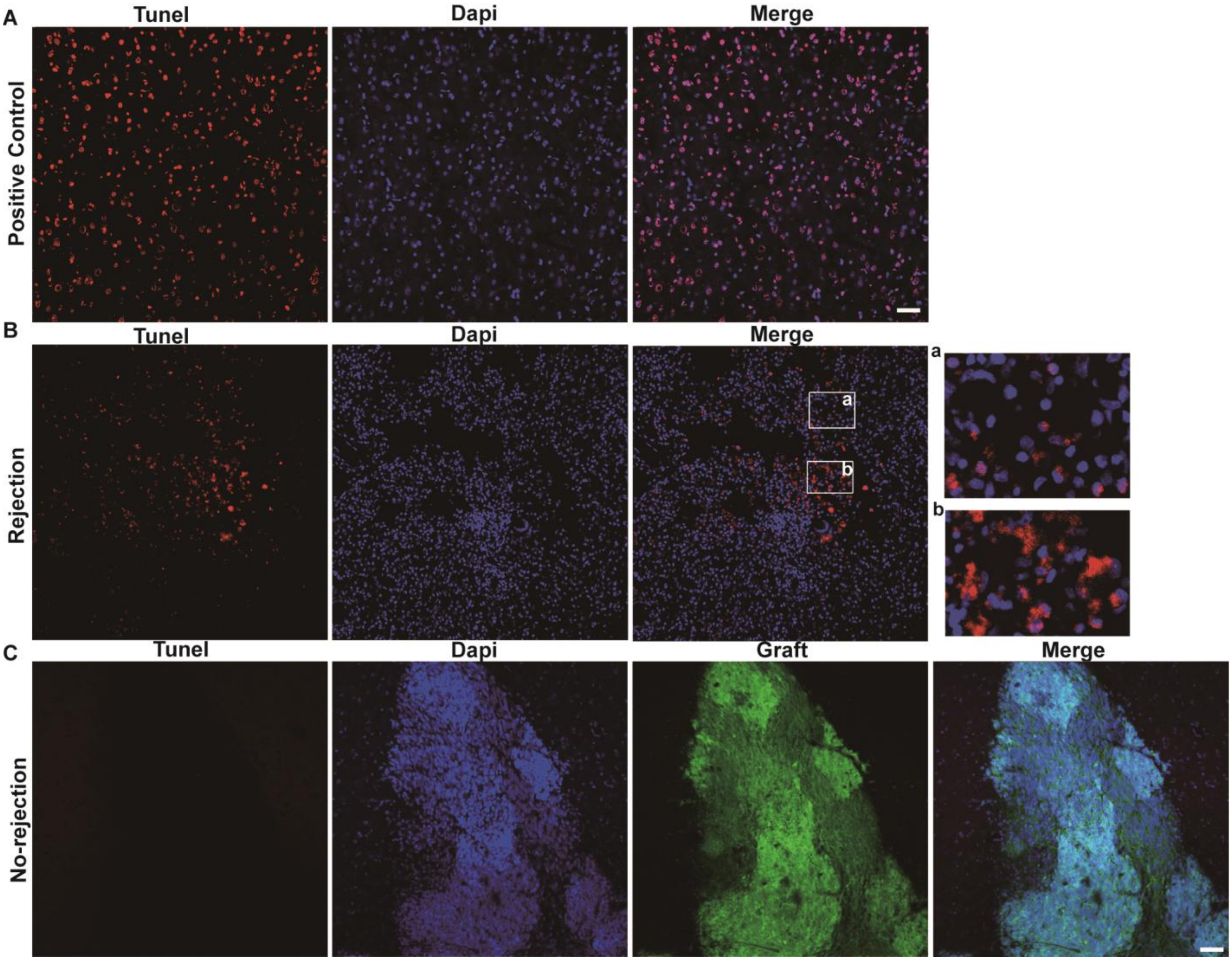

Figure S4 TUNEL assay to confirm the apoptosis/necrosis process within the rejected graft. A, brain slice treated with DNAase I served as the positive control. B, TUNEL signals detected within the rejected graft region. B-a showing TUNEL signals within the nucleus; B-b showing TUNEL signals within the cytoplasm. C, the nearly absence of TUNEL signals in non-rejected graft. Scale bar, 50 µm.

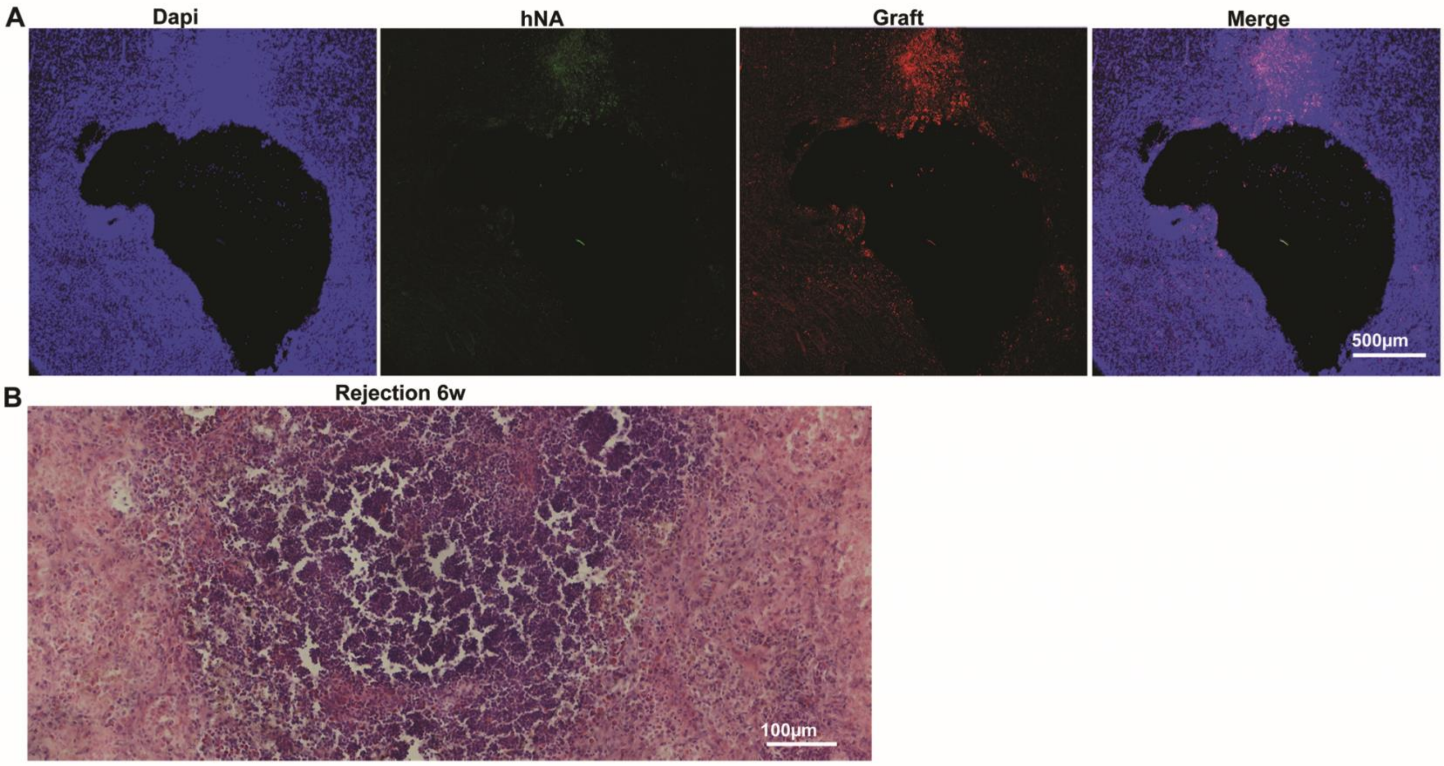

Figure S5. The massive death of human NPC grafts is due to immuno-rejection in the vast majority of cases, related to Figure 3. A, conspicuous cavity found in some rejection cases. B, H&E staining showing heavy leukocyte infiltration with absurd cracks within the rejected graft area.

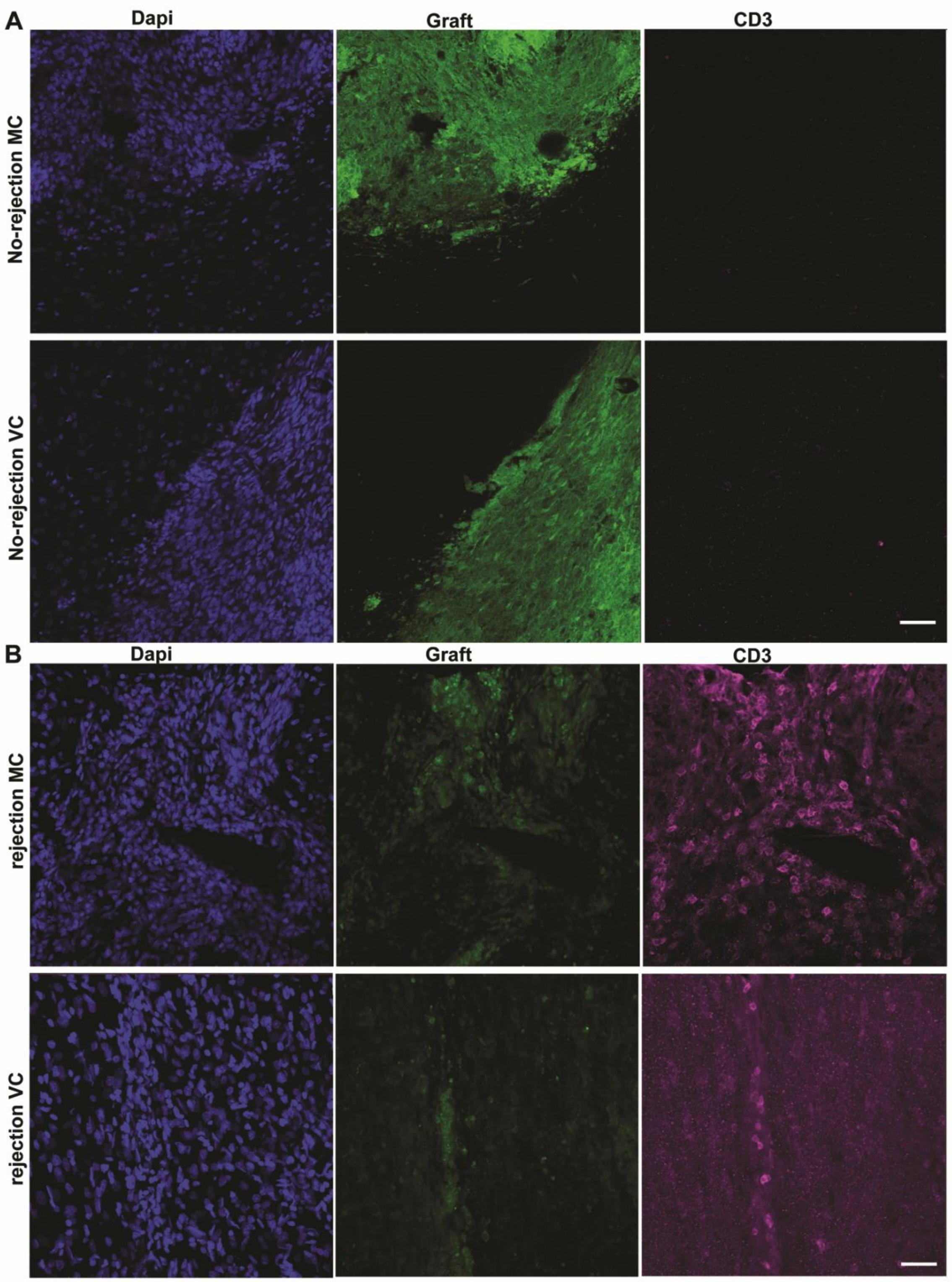

Figure S6. Two hNPC grafts in different sites of the same rats are rejected in a "both or none" manner. A, showing both grafts survived without CD3+ lymphocyte infiltration. B, showing both grafts rejected accompanied by CD3+ lymphocyte infiltration. Scale bar, 50 μm.

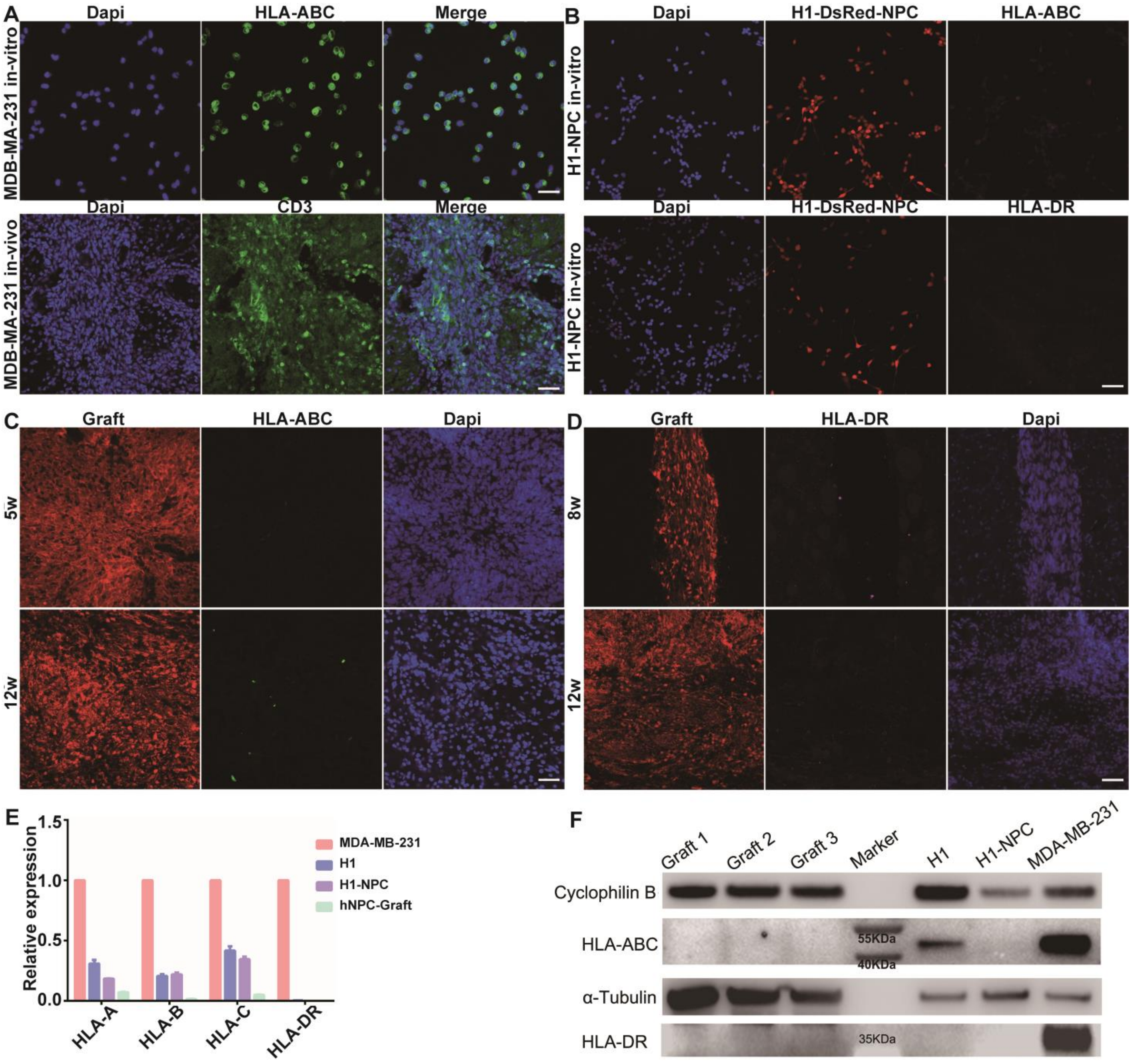

Figure S7. Human NPC derivates retain low HLA antigen levels, accounted for long-term survival. A, human MDB-MA-231 cells express high-level HLA-ABC (UP), and are rejected quickly after transplantation, accompanied by CD3+ lymphocyte infiltration (Bottom). B-D, human NPC derivates retain low HLA expression levels both *in vitro* (B) and *in vivo* over time (C,D). Scale bar, 50 µm. E, the mRNA expression levels of HLA antigens of the H1 cells and derivates relative to MDB-MA-231 cells. F, the protein levels of HLA antigens of H1 cells and derivates determined by Western Blot. H1: H1 ESCs; H1-NPC: H1 ESC-derived NPCs; hNPC graft or graft: grafted H1 ESC-derived NPCs.

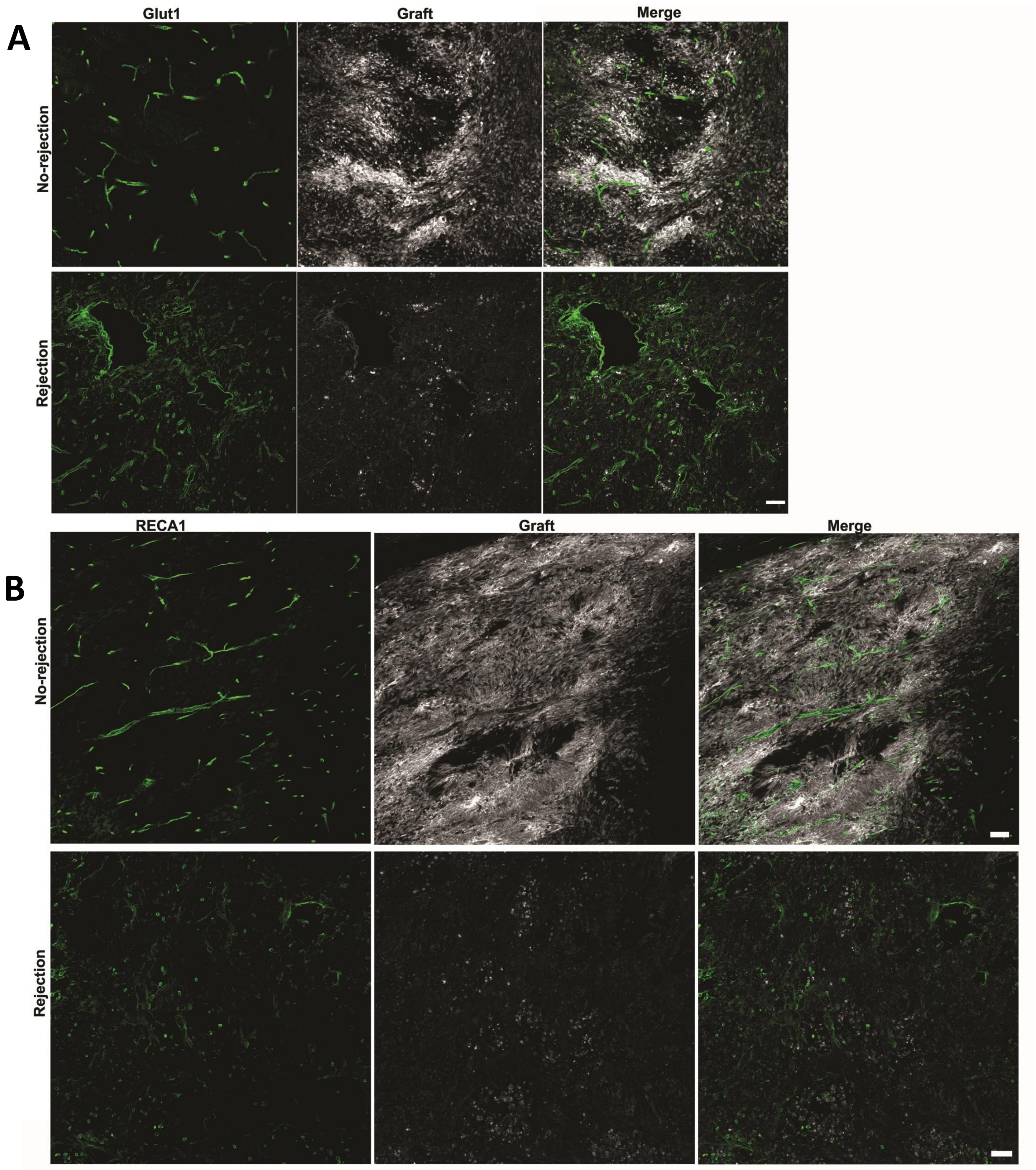

Figure S8. Immunostaining of vascular markers Glut1 (A) and ReCA1(B) showing that the collapses of the local microvessels within the rejected grafts, while the blood vessels in the non-rejected grafts stay in good morphology.

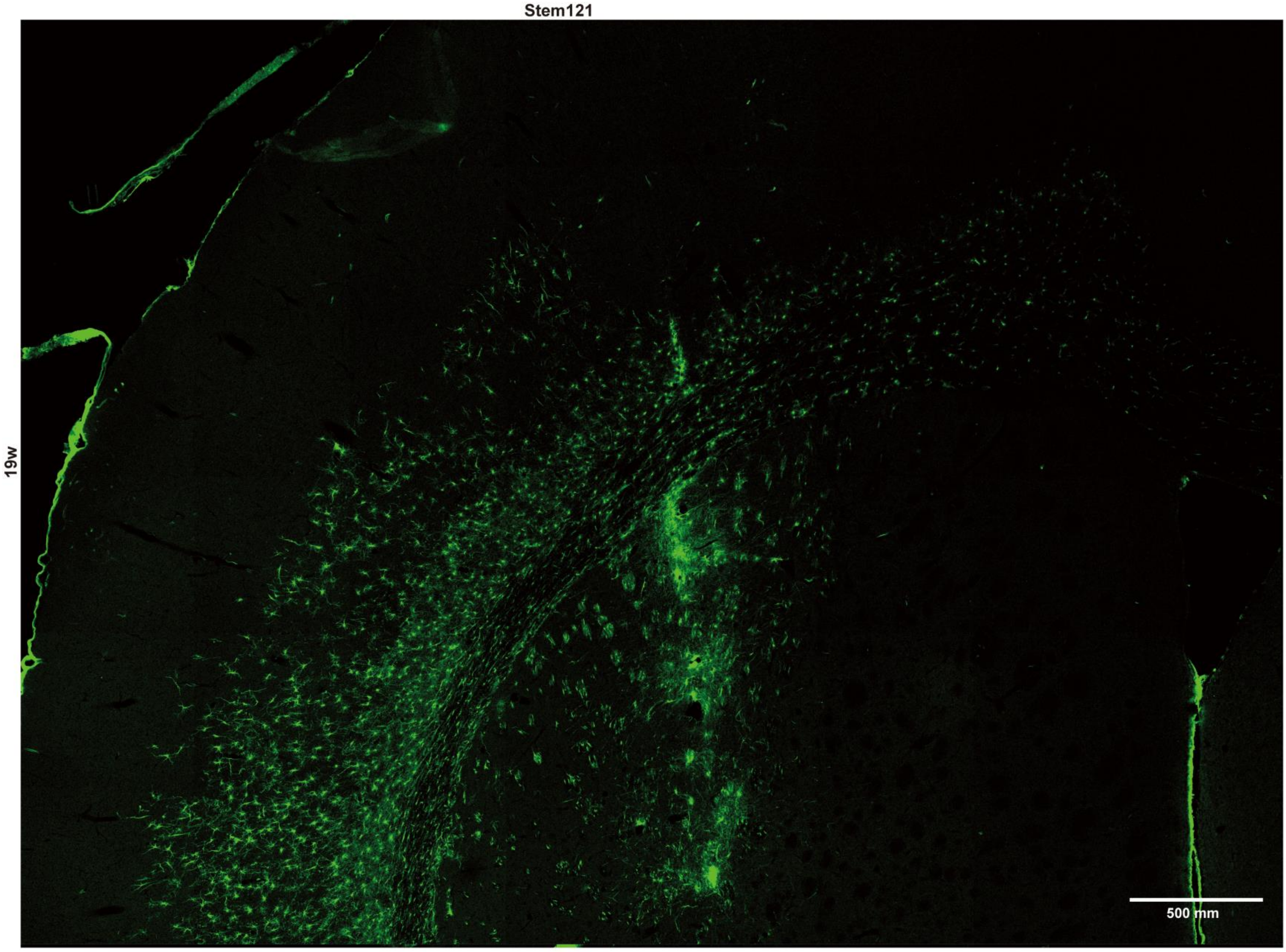

Figure S9. A representative brain slice showing wide migration of human late passaged NPC into the host cerebral cortex without apparent graft core at 19 wpt.

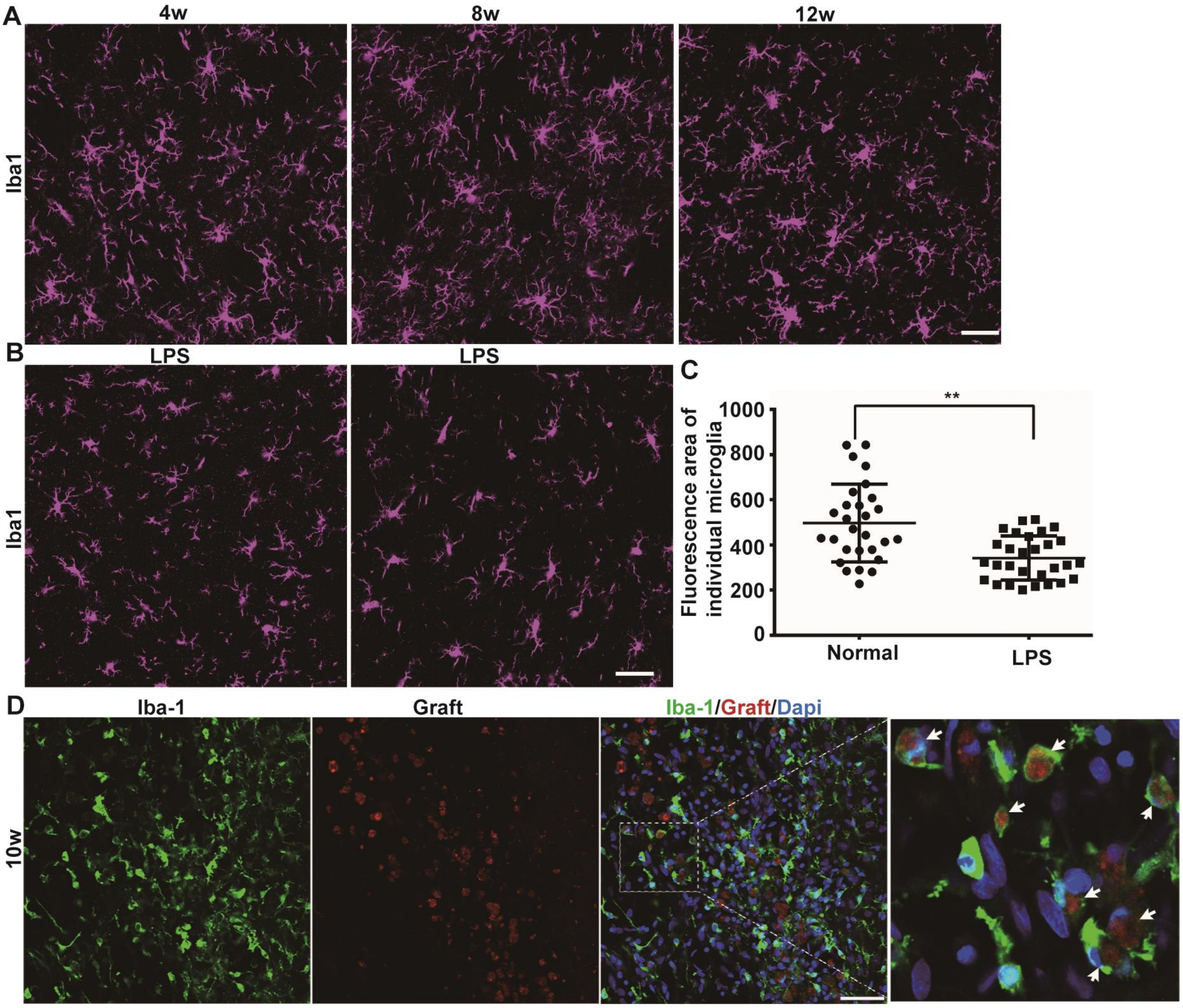

Figure S10. Representative pictures showing the different states of microglia. A, the resting microglia in the grey matter of the contralateral hemisphere display a tiling and ramified profiling with long, thin and highly branched processes. B, Mildly activated microglia by LPS challenge in non-graft area show retracted and thick processes C, statistical analysis of the cover area by a single microglia between resting and mildly activated state. D, microglia in the rejected graft area show typical phagocytosing shape with DsRed signals found inside the cytoplasm of microglia (indicated by arrows). Scale bar, 50 µm.

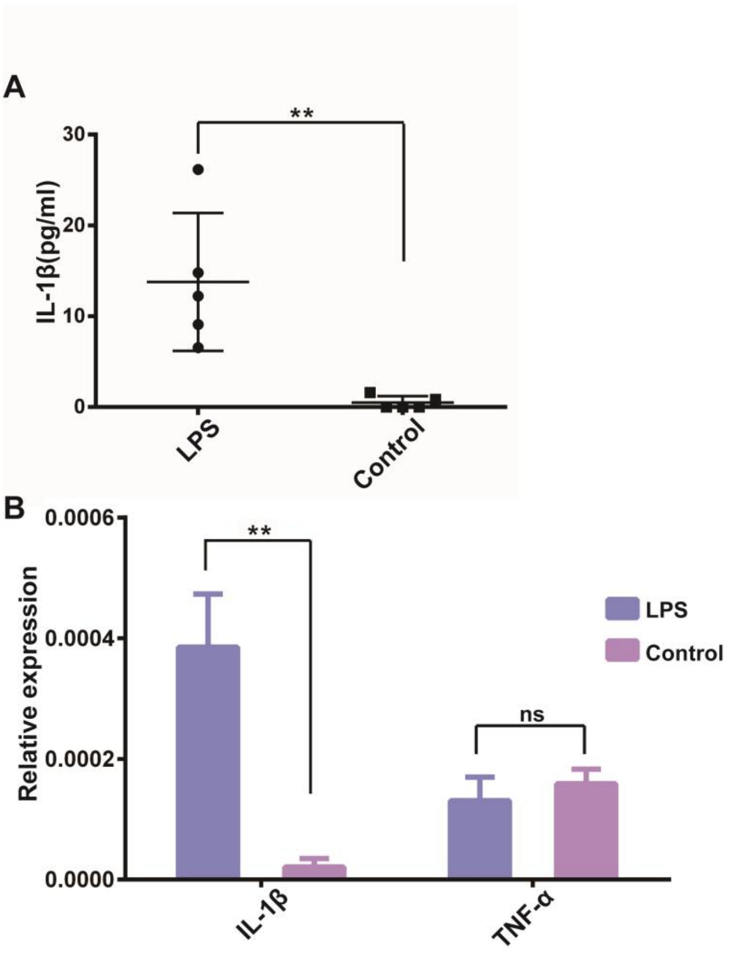

Figure S11. LPS challenge significantly increases the secretion of inflammation cytokine IL-1β in the brain at 24h after the 2nd injection. A, ELISA for soluble IL-1β. The soluble TNFα level was also examined but under the detection level. B, the relative mRNA expression levels of IL-1β and TNFα.

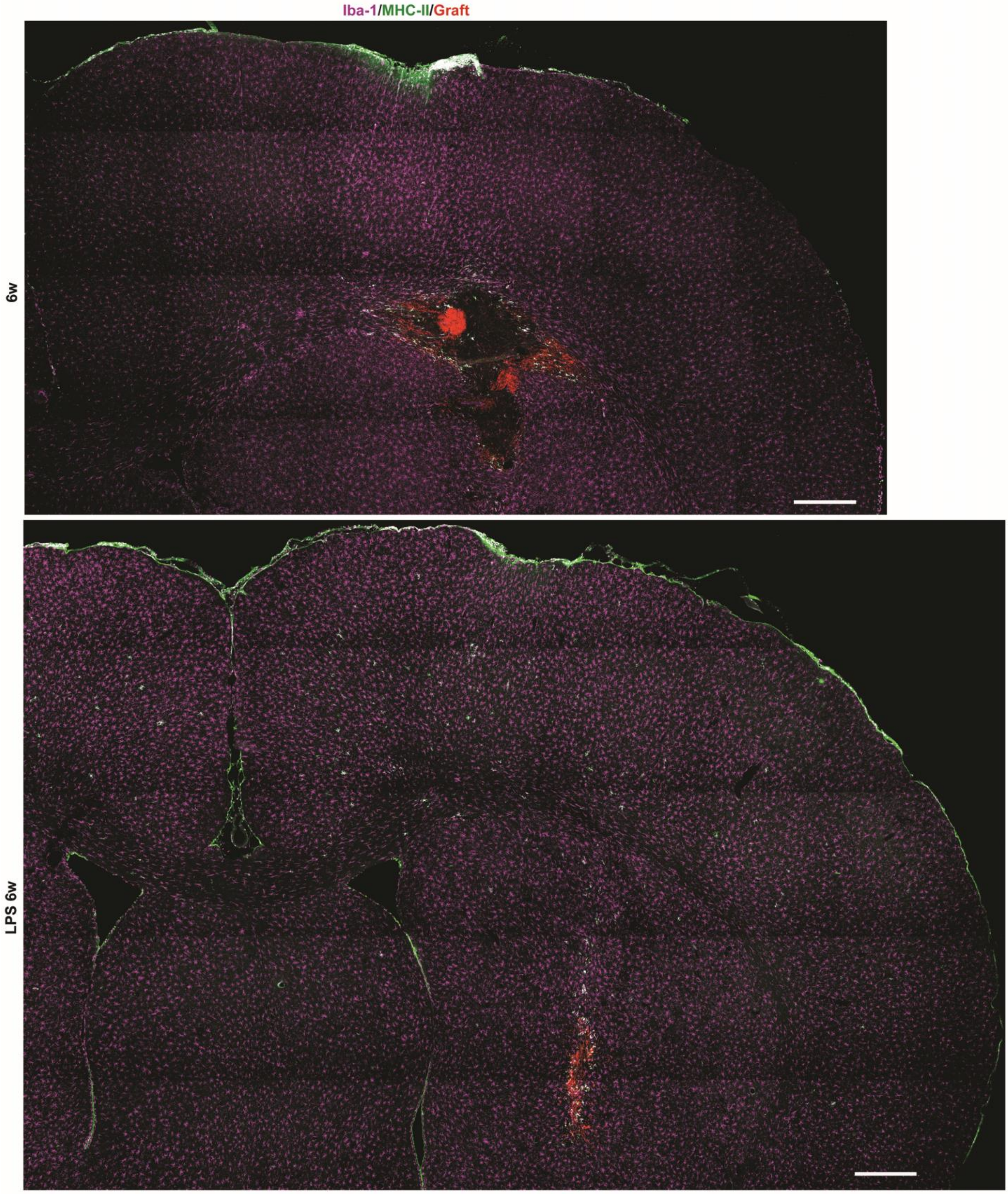

Figure S12. LPS induces a brain-wide activation of microglia with sparsely increased MHC-II expression at 10 days later. Scale bar, 500 µm.
